# The Genus *Picoa* (Pyronemataceae, Pezizales) Revisited

**DOI:** 10.3390/jof12020084

**Published:** 2026-01-27

**Authors:** Pablo Alvarado, Aurelia Paz, Claude Lavoise, Nicolas Van Vooren

**Affiliations:** 1 Independent Researcher, ALVALAB, Dr. Fernando Bongera St., Severo Ochoa Bldg. S1.04, 33006 Oviedo, Spain; 2 Independent Researcher, VallTer 791, Urbanització Llac del Cigne, 17455 Caldes de Malavella, Spain; 3 Independent Researcher, 14A Rue Professeur Deperet, 69160 Tassin-la-Demi-Lune, France; nvanvooren@msn.com

**Keywords:** Cistaceae, *Geopora*, hypogeous fungi, Mediterranean basin, *Sepultaria*, taxonomy

## Abstract

The genus *Picoa* is here revisited after obtaining new molecular and morphological data. Morphological features of type material and other historical collections of *P. juniperi* and *P. lefebvrei* were studied and compared with recent collections obtained from several European, African, and Asian countries. Genetic studies employing ITS rDNA, as well as 28S rDNA and the RPB2 gene, were conducted on modern samples. As a result, 19 taxa are identified, 17 of which are new to science. Detailed descriptions of all species are provided, including macro- and micro-photographs, and an identification key. The phylogenetic structure of the genus is discussed, and the main clades are described in the following new sections: *Picoa* sect. *Communes*, sect. *Juniperi*, sect. *Lefebvreorum*, sect. *Microsporae*, and sect. *Puenteorum*. Finally, the boundaries of the genus *Picoa* are evaluated by comparison with the sister genera *Geopora*, *Sepultaria*, and *Terracavicola*.

## 1. Introduction

The genus *Picoa* Vittad. was published in 1831 by Carlo Vittadini in his work “Monographia Tuberacearum” [1]. The genus was dedicated to Vittorio Pico for his work *Melethemata* [2] and his overall contribution to mycology. *Picoa* was originally proposed for a single species, *P. juniperi* Vittad., first collected in northern Italy, near *Juniperus* sp. Vittadini [1] reported that *P. juniperi* had globose ascomata with a black and warty surface; a white gleba full of pseudoveins; globose asci; and smooth, globose ascospores. Later, in 1894, Patouillard [3] proposed the genus *Phaeangium* Pat. for *Ph. lefebvrei* Pat., a species first collected at Ras-el-Oued (Tunisia), characterized by globose to subglobose, brownish ascomata; surface covered by many hairs; and a white gleba inside and, microscopically, by stipitate asci and smooth ovoid ascospores. Some years later, Maire [4] combined this species as *Picoa lefebvrei* (Pat.) Maire.

*Picoa carthusiana* Tul. & C. Tul. was published in 1862 by Tulasne & Tulasne [5], but it was later combined in the genus *Leucangium* Quél. by Saccardo [6]. O’Donnell et al. [7] confirmed this decision after the analysis of SSU and LSU rDNA sequences and found that this genus is related to the families *Morchellaceae* and *Helvellaceae*. In 1956, Lange [8] published *P. pachyascus* M. Lange, a North American species, but it was later synonymized with *Imaia gigantea* (S. Imai) Trappe & Kovács by Kovács et al. [9]. One last species, *P. melospora* G. Moreno, J. Díez & Manjón [10], was published in 2000 but later combined in the genus *Tuber* P. Micheli ex F.H. Wigg. as *T. melosporum* (G. Moreno, J. Díez & Manjón) P. Alvarado, G. Moreno, Manjón & J. Díez [11].

The first molecular analyses, published by Gutiérrez et al. [12], suggested that *Picoa* belongs in the family *Pyronemataceae*, being closely related to the genus *Geopora* Harkn. Zitouni-Haouar et al. [13] analyzed samples from multiple Mediterranean countries, using SEM to study the ornamentation of the ascospores (including some historical collections of *P. juniperi* and *P. lefebvrei*). They identified six major lineages in *Picoa,* apparently associated with different host plants and climatic preferences. The authors suggested that these factors could have driven the speciation process of *Picoa*. However, the color and shape of the ascomata and spore ornaments were not considered sufficient to reliably discriminate *P. juniperi* and *P. lefebvrei*.

In the present work, morphological and genetic data obtained from new samples of *Picoa* were compared with the previously known information, aiming to fix the identity of the type species, *P. juniperi*; identify new diagnostic morphological features to discriminate the most frequent phylogenetic clades; and propose new names for those lacking them.

## 2. Materials and Methods

### 2.1. Fungal Collections

The holotypes are preserved in the CeDocBiV herbarium of the University of Barcelona (BCN). Isotypes and other collections are deposited at the herbarium of the University of Alcalá (AH), as well as the personal herbaria of A. Paz (IC), B. Moreno-Arroyo (BM), F. Sáinz (PSS), J. Gómez (HB), Asociación Vallisoletana de Micología (AVM), L. A. Trujillo (LTP), M. A. Ribes (MAR), and G. Konstantinidis (GK). Fungal collections of M. Slavova and B. Assyov were deposited at the Mycological Collection of the Research Institute of Biodiversity and Ecosystems of the Bulgarian Academy of Sciences (SOMF).

### 2.2. Morphological Study

Colors of ascomata were recorded from fresh specimens. For each collection, available data on its collection site, habitat, collection date, collector, and herbarium number are indicated. The term “calcareous soils” refers to basic soils (pH 7.2–8.4) that are supposed to contain a large amount of calcium carbonate, while “neutral soils” refers to neutral soils (pH 6.5–7.2) that are supposed to have balanced percentages and availability of primary and secondary chemical elements. When two or more collections were reported from the same locality, successive collections were labeled “Ibid.”, indicating only the data that vary in relation to the first sample. Sequenced collections are marked with the symbol “*”.

Microscopic features were studied with a Nikon Eclipse E800 microscope (Nikon Corporation, Tokyo, Japan), equipped with planar apochromatic optics, coupled to a Nikon D7100 reflex camera (24.1 Mpx). Microscopic studies were conducted on fresh material or dried herbarium material (in the last case, a small amount was rehydrated with water for 24 h). For ascospore comparisons, permanent slides on Hoyer’s medium were prepared from all collections. The ascospores were measured in lateral view (length × width), including the perisporium and ornamentation. At least 10 series of 20 ascospores were measured from each specimen studied, indicating the minimum, mean (in italics), and maximum measurements and the minimum and maximum Q values. Measurements of ascospores and other cells were taken with the “Mycomètre VA 2.07” software (G. Fannechère), based on methods proposed by Fannechère [14]. Light microscopy images of ascospores were digitally stacked with the “Helicon Focus 8.3.0” software (Helicon Soft Ltd., Kharkov, Ukraine).

### 2.3. DNA Extraction, PCR Amplification, and Sequencing

Total DNA was extracted from dry specimens employing a modified protocol based on that of Murray and Thompson [15]. PCR reactions [16] included 35 cycles with an annealing temperature of 54 °C. The primers ITS1F and ITS4 [17,18] were employed to amplify the ITS rDNA region, LR0R and LR5 [19,20] were used for the 28S rDNA region, and bRPB2-6F2 (bRPB2-6R2 in forward direction) and bRPB2-7R2 were used for the RNA polymerase II second-largest subunit (RPB2) gene [21]. PCR products were checked in 1% agarose gels, and amplicons were sequenced with one or both PCR primers. Sequences were manually corrected to remove reading errors in chromatograms.

### 2.4. Phylogenetic Analyses

Two datasets were built: (1) a multigene alignment including ITS rDNA, 28S rDNA, and RPB2 sequences from selected specimens of *Picoa* and related genera (*Geopora*, *Sepultaria*, and *Terracavicola*), with *Tricharina* as the outgroup, according to ITS and LSU analyses in Grupe II et al. [22]; and (2) an alignment including all ITS rDNA sequences of *Picoa* with selected ITS sequences of *Geopora* as the outgroup. BLASTn algorithm in BLAST+ 2.17.0 [23] was used to select the most closely related sequences from the International Nucleotide Sequence Database Collaboration public database (INSDC) [24] and UNITE [25]. The sequences retrieved came mainly from Zitouni-Haouar et al. [13]. Sequences in each dataset (Supplementary Table, S1) were first aligned in MEGA 5.0 [26] with its Clustal W application [27] and then realigned manually as needed to establish positional homology (i.e., looking for broken motifs present in sequences with high similarity).

Aligned datasets (Supplementary Files S1 and S2) were subjected to MrModeltest 2.3 [28] in PAUP* 4.0b10 [29]. The GTR + G + I model was selected and implemented in MrBayes 3.2.6 [30], where a Bayesian analysis was performed. Dataset 1 was divided into 3 partitions (ITS, LSU, and RPB2), while Dataset 2 included a single partition (ITS). The analysis comprised two simultaneous runs, four chains, a temperature set to 0.2, and sampling every 100th generation (excluding the first 25% as burn-in) until the average split frequencies between the simultaneous runs fell below 0.01, which happened after 1.35 M generations (ITS dataset) and 0.37 M generations (multigene dataset). Finally, a full search for the best-scoring maximum likelihood tree was performed in RAxML 8.2.12 [31] using the standard search algorithm (same partitions, GTRGAMMAI model, 2000 bootstrap replications). The significance threshold was set ≥0.95 for posterior probability (PP) and ≥70% bootstrap percentage (BP).

## 3. Results

### 3.1. Phylogenetic Results

**Figure 1:**
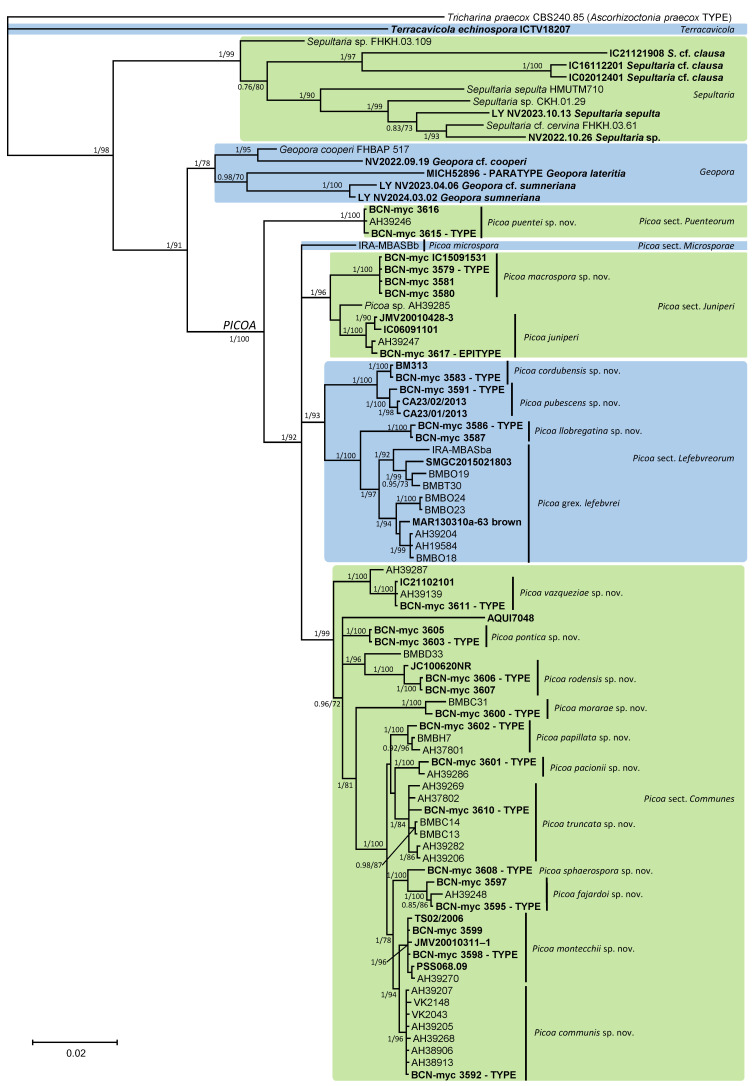
A 50% majority rule ITS—28S rDNA—RPB2 consensus phylogram of *Picoa* and related genera (with *Tricharina praecox* as outgroup) obtained using MrBayes from 2775 sampled trees. Nodes were annotated if they were supported by ≥0.95 Bayesian posterior probability (**left**) or ≥70% maximum likelihood bootstrap proportions (**right**). Sequences newly generated in this study are in bold.

**Figure 2:**
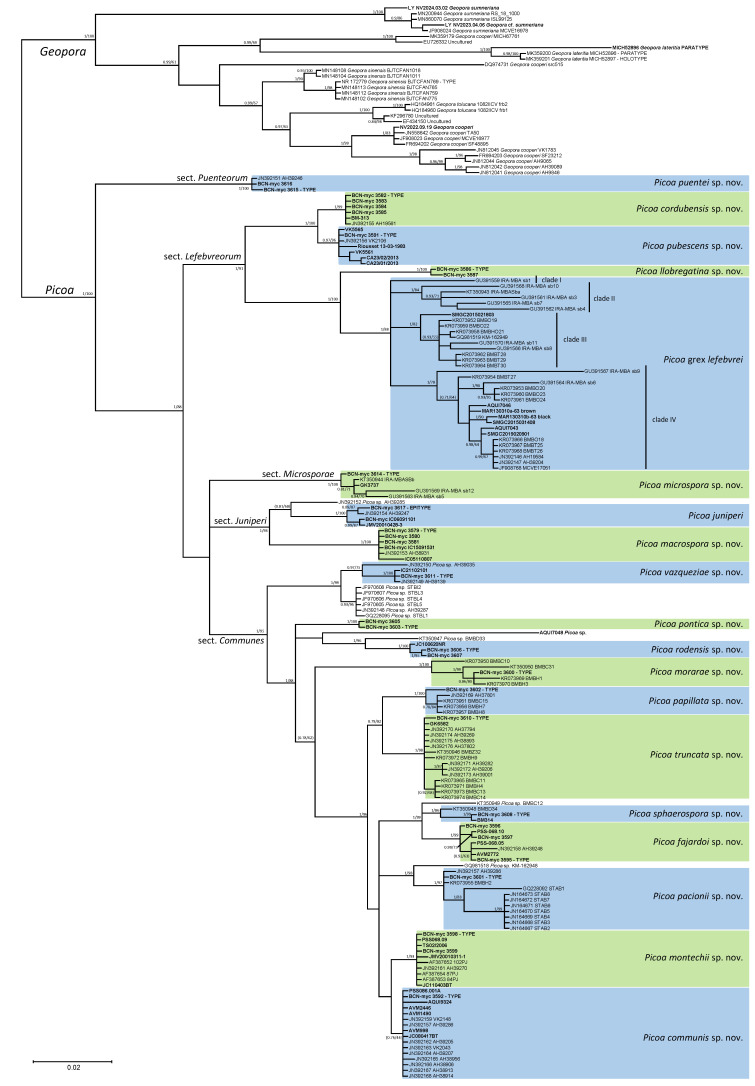
A 50% majority rule ITS rDNA consensus phylogram of *Picoa* and *Geopora* obtained using MrBayes from 10,125 sampled trees. Nodes were annotated if they were supported by ≥0.95 Bayesian posterior probability (**left**) or ≥70% maximum likelihood bootstrap proportions (**right**). Nonsignificant support values (≥0.90 PP or ≥60% BP) are represented inside parentheses. Sequences newly generated in this study are in bold.

The phylogenetic analyses of the ITS, LSU, and RPB2 sequences of *Picoa* and related genera (Figure 1) showed that all samples of *Picoa* form a monophyletic clade sister to the genus *Geopora* (type: *G. cooperi* Harkn.), which also contains species producing sequestrate hypogeous or semi-epigeous ascomata, such as *G. lateritia* (Fogel & States) Healy & M.E. Sm. and *G. sumneriana* (Cooke ex W. Phillips) M. Torre. The genus *Sepultaria* (Cooke) Boud. (type: *Peziza sepulta* Fr.), which also includes sequestrate species (i.e., *Geopora clausa* (Tul. & C. Tul.) Burds.), seems unrelated to *Geopora*. The recently discovered sequestrate genus *Terracavicola* A.C. Grupe, Kraisit., Guevara & M.E. Sm. (type: *T. echinospora* A.C. Grupe, Kraisit., Guevara & M.E. Sm.), which was found to be distantly related to *Picoa*, *Geopora*, and *Sepultaria* in previous phylogenies based on rDNA data [22], does not show significant similarities in the present analyses, also using RPB2 sequences. The multigene analysis found that *Picoa* is composed of at least five major clades, which are here organized into new sections (see under Taxonomy): *Picoa* sect. *Puenteorum* (basal to all others), sect. *Lefebvreorum*, sect. *Microsporae*, sect. *Juniperi*, and sect. *Communes*. The extensive analysis of the ITS data of *Picoa* (Figure 2) produced a similar infrageneric topology.

On the basis of both the morphological and molecular analyses, the genus *Picoa* is here thought to include at least 19 species, which are described below. Six unnamed clades very likely represent also new species that should be properly studied with representative material. The clade named *Picoa* grex *lefebvrei* here might also include up to four different species, but their status is not addressed in the present work because of the incomplete data available from them.

### 3.2. Taxonomy

**Figure 3:**
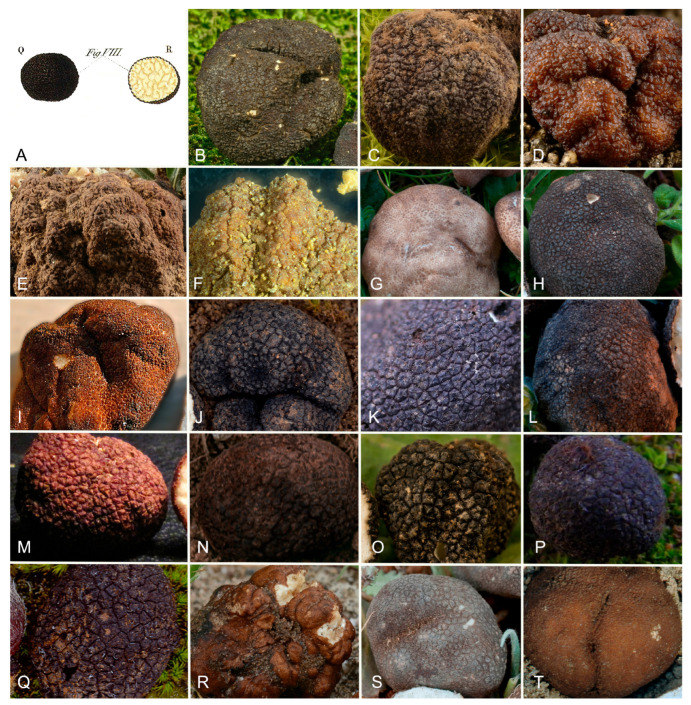
Comparative ascomata surface of *Picoa*: (**A**) *P. juniperi* (lectotype); (**B**) *P. communis* (holotype); (**C**) *P. montecchii* (holotype); (**D**) *P. sphaerospora* (holotype); (**E**) *P. fajardoi* (holotype); (**F**) *P. pacionii* (holotype); (**G**) *P. papillata* (holotype); (**H**) *P. truncata* (holotype); (**I**) *P. morarae* (holotype); (**J**) *P. vazqueziae* (holotype); (**K**) *P. pontica* (holotype); (**L**) *P. rodensis* (holotype); (**M**) *P. microspora* (holotype); (**N**) *P. juniperi* (BCN-myc 3617); (**O**) *P. macrospora* (holotype); (**P**) *P. pubescens* (holotype); (**Q**) *P. cordubensis* (holotype); (**R**) *P. llobregatina* (holotype); (**S**) *P. lefebvrei* (MAR130310-63a brown); (**T**) *P. puentei* (holotype). Photos: A. Paz. All photo images were taken from fresh material, except (**F**,**M**), which were taken from dry specimens.

**Figure 4:**
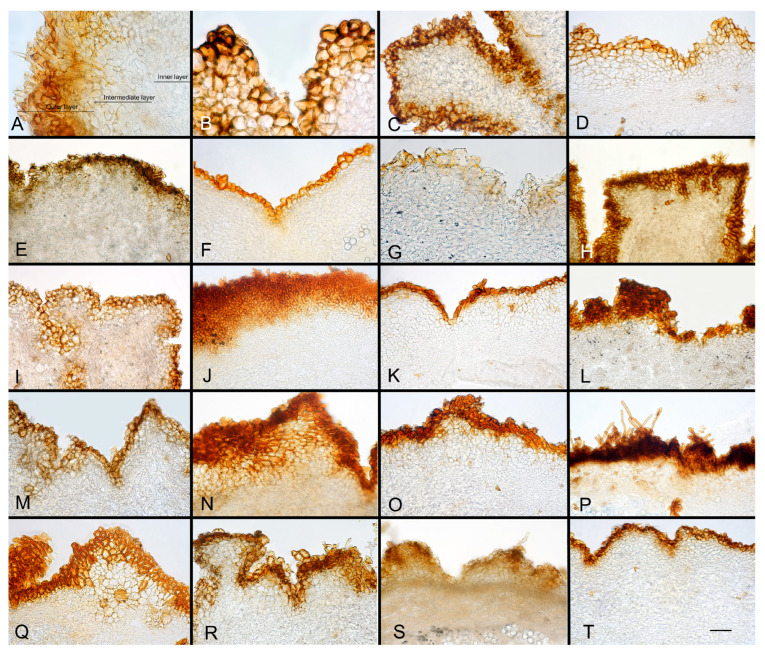
Comparative peridium sections of *Picoa*: (**A**) Peridium structure in genus *Picoa*; (**B**) *P. communis* (holotype); (**C**) *P. montecchii* (holotype); (**D**) *P. sphaerospora* (holotype); (**E**) *P. fajardoi* (holotype); (**F**) *P. pacionii* (holotype); (**G**) *P. papillata* (holotype); (**H**) *P. truncata* (holotype); (**I**) *P. morarae* (holotype); (**J**) *P. vazqueziae* (holotype); (**K**) *P. pontica* (holotype); (**L**) *P. rodensis* (holotype); (**M**) *P. microspora* (holotype); (**N**) *P. juniperi* (Herbier Général Champignons PC0022113); (**O**) *P. macrospora* (holotype); (**P**) *P. pubescens* (holotype); (**Q**) *P. cordubensis* (holotype); (**R**) *P. llobregatina* (holotype); (**S**) *P. lefebvrei* (col. Maire, M 157945); (**T**) *P. puentei* (holotype). Scale bar = 40 μm. Photos: A. Paz. All images were taken from fresh material, except (**F**,**I–K**,**M**,**N**,**S**,**T**) (rehydrated).

**Figure 5:**
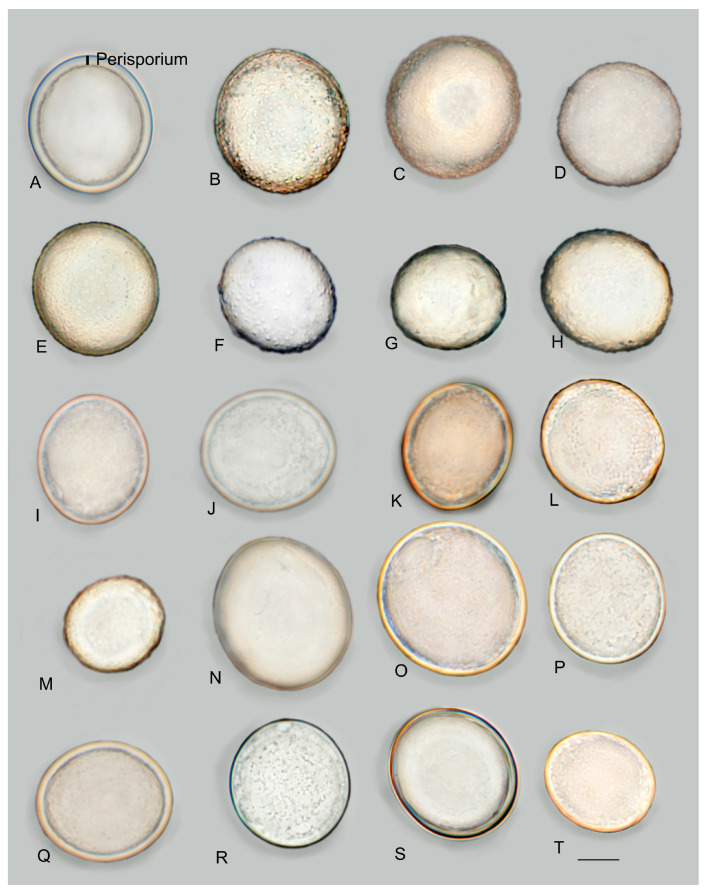
Comparative ascospores of *Picoa*: (**A**) Spore structure in genus *Picoa*; (**B**) *P. communis* (holotype); (**C**) *P. montecchii* (holotype); (**D**) *P. sphaerospora* (holotype); (**E**) *P. fajardoi* (holotype); (**F**) *P. pacionii* (holotype); (**G**) *P. papillata* (holotype); (**H**) *P. truncata* (holotype); (**I**) *P. morarae* (holotype); (**J**) *P. vazqueziae* (holotype); (**K**) *P. pontica* (holotype); (**L**) *P. rodensis* (holotype); (**M**) *P. microspora* (holotype); (**N**) *P. juniperi* (Herbier Général Champignons PC0022113); (**O**) *P. macrospora* (holotype); (**P**) *P. pubescens* (holotype); (**Q**) *P. cordubensis* (holotype); (**R**) *P. llobregatina* (holotype); (**S**) *P. lefebvrei* (coll. Maire, M 157945); (**T**) *P. puentei* (holotype). Scale bar = 10 μm. Photos: A. Paz. All images were taken from fresh material, except (**F**,**I**–**K**,**M**,**N**,**S**,**T**) (rehydrated).

1. ***Picoa*** Vittad., Monogr. Tuberac.: 54. 1831. MB 4096. Figure 3, Figure 4 and Figure 5.
  The genus *Picoa* is characterized by having globose to subglobose or sometimes slightly lobed ascoma, compact gleba, with irregularly arranged fertile cells and, microscopically, by asci inamyloid, prototunicate, subglobose, with a long and simple thin-walled peduncle, containing 4–8 ascospores, spherical to ellipsoidal, hyaline, or slightly pigmented in golden tones, ornamented with granules, rugosities, or ridges when fully mature. The species grow under *Cassia*, *Cistus*, *Cytisus*, *Helianthemum*, *Quercus*, and/or *Tuberaria* in arid, sandy, neutral to calcareous soils.
  *Type*: *Picoa juniperi* Vittad.
  *Notes*: In the present work, the genus *Picoa* is formed by at least five main clades, here defined as sections. They roughly correspond to the major lineages already found by one of the authors (PA) in Zitouni-Haouar et al. [13], although lineages II and III in that work are merged into a single section (sect. *Lefebvreorum*) in the present study. The genus *Picoa* is sister to *Geopora* s. str. Both genera have globose or subglobose apothecia, an external surface (in most species) with warts and mycelial hairs, a distinctive hymenium, and sometimes a very folded gleba and, microscopically, inamyloid asci (staining blue in Melzer reagent), hyaline and cylindrical paraphyses, and mostly ellipsoidal to broadly ellipsoidal ascospores. The genus *Sepultaria* is also phylogenetically related to *Picoa*. It contains epigeous and hypogeous species and resembles *Picoa* because of its often warty external surface covered with mycelial hairs, distinct hymenium, inamyloid asci, hyaline paraphyses, and ellipsoid to broadly ellipsoid ascospores.

**Key to the sections of *Picoa***

1.Commonly fruiting in summer or autumn, under *Quercus* (with or without presence of *Juniperus*), in Mediterranean Europe, dark brown to black ascomata, ascospores > 28 µm long.*Picoa* sect. *Juniperi.*1.Commonly fruiting in late winter and in spring, under Cistaceae with or without tree hosts, in the European and African Mediterranean shores, Canary Islands, and the Levant and Middle East; variable ascomata; ascospores < 28 µm long.22.Ascospores 22 × 19 µm on average.32.Ascospores 25.5 × 22.5 µm on average.43.External surface covered with pyramidal warts, by now found in Mediterranean Africa and the Levant, associated with perennial *Helianthemum*; ascospores ornamented with coarse granules scattered on a rugose surface.*Picoa* sect. *Microsporae*.3.External surface covered with granules, by now found only in central Spain, associated with different Cistaceae and tree hosts; ascospores ornamented with minute granules that coalesce to form irregular ridges, with a thick, golden-orange perispore.*Picoa* sect. *Puenteorum.*4.Mostly associated with perennial Cistaceae (*Helianthemum*, *Cistus*, *Fumana*), in sandy neutral soil, ascospores broadly ellipsoidal, up to 27 µm long, ornamented with fine granules that join together to form ridges or semi-reticles.*Picoa* sect. *Lefebvreorum.*4.Mostly associated with annual *Helianthemum* (but maybe also *Cistus* and other hosts), in variable soils, ascospores globose to ellipsoidal, up to 27 µm long, ornamented with isolated granules that rarely form a reticulum.*Picoa* sect. *Communes.*

**Figure 6:**
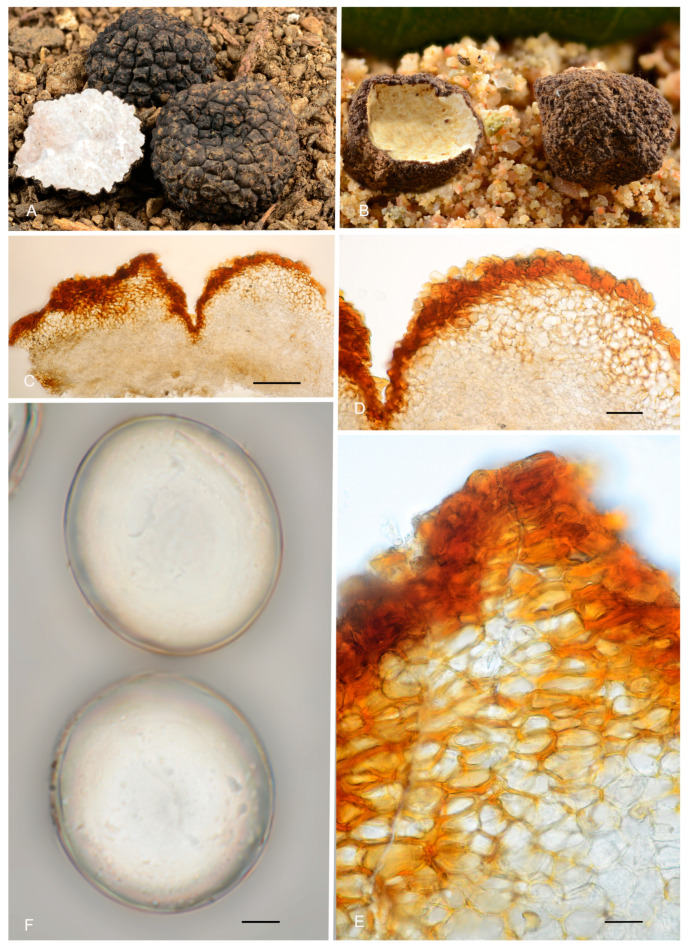
*Picoa juniperi*: (**A**) ascomata IC06091101; (**B**) ascomata AZ. 1404, BCN-myc 3617; (**C**–**F**) Herbier Général Champignons PC0022113: (**C**) section of peridium and gleba; (**D**,**E**) medullary excipulum with pseudoparenchymatous structure; (**F**) ascospores. Scale bars: (**C**) = 2 mm; (**D**) = 40 µm; (**E**) = 20 µm; (**F**) = 5 µm. Photos: A. Paz. Images (**A**,**B**) taken from fresh material; (**C**–**F**) taken from rehydrated material.

**Figure 7:**
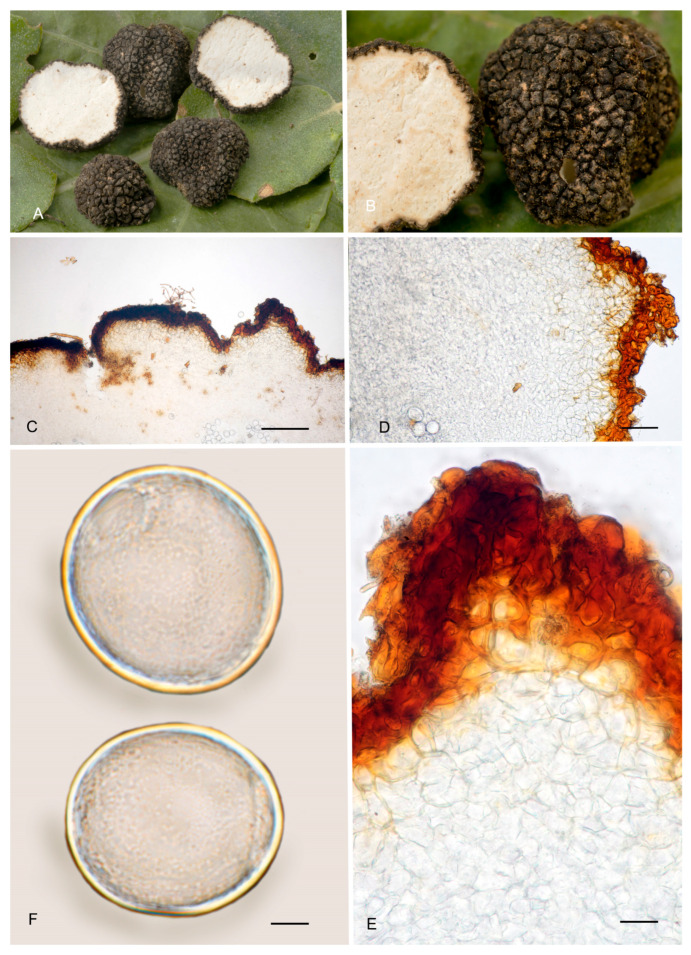
*Picoa macrospora* BCN-myc 3579 (holotype): (**A**,**B**) ascomata; (**C**) section of peridium and gleba; (**D**,**E**) medullary excipulum with pseudoparenchymatous structure, made of subglobose or slightly elongated sclerenchymatous cells; (**F**) ascospores. Scale bars: (**C**) = 2 mm; (**D**) = 40 µm; (**E**) = 20 µm; (**F**) = 5 µm. Photos: (**A**,**B**) = R. Martinez; (**C**–**F**) = A. Paz. All images were taken from fresh material.

2. ***Picoa*** **sect.**  ***Juniperi*.** Figure 3, Figure 4, Figure 5, Figure 6 and Figure 7.
  *Type*: *Picoa juniperi* Vittad.
  *Notes*: *Picoa* sect. *Juniperi*, which contains the type of *Picoa*, is characterized by producing dark brown or black ascomata covered with compound warts, sclerenchymatous cells in the outer layers, ellipsoidal ascospores measuring more than 28 µm long, ornamented with isolated coarse or very fine granules that coalesce to form incomplete reticles. Species of the section *Juniperi* seem to occur in calcareous soils, probably associated with *Quercus* and/or *Juniperus*. Two species belong to this section, *P. juniperi* and *P. macrospora*, but a third one likely exists (lineage of AH 39285).

**Key to the sections of *Picoa* sect. *Juniperi***

1.Ascomata with a pseudoparenchymatous internal layer of the peridium, ascospores broadly ellipsoidal, 28 × 25.5 µm on average, ornamented with thick, isolated granules over a slightly rough surface.
*P. juniperi.*
1.Ascomata with an intricate plectenchymatous internal layer of the peridium, ascospores broadly ellipsoidal, 31 × 28 µm on average, ornamented with very fine, not very pronounced granules, which join together at the bases to form semi-reticles.
*P. macrospora.*

**Figure 8:**
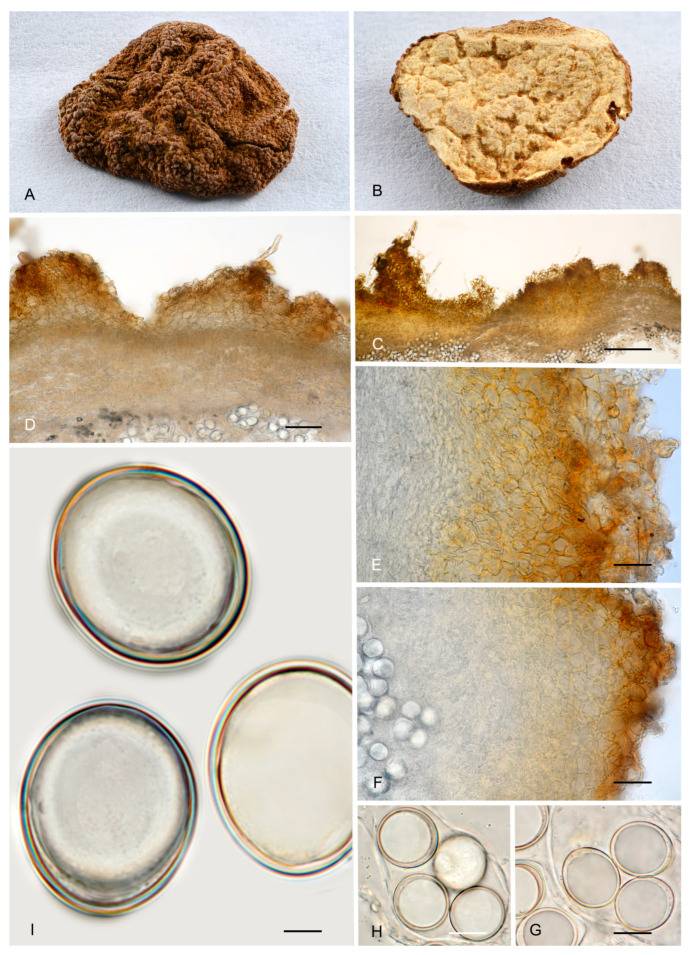
*Picoa lefebvrei* (**A**,**B**) coll. Maire, M 157945: (**A**) ascomata; (**B**) detail of gleba; (**C**–**I**) FH 301557 (holotype): (**C**,**D**) section of peridium and gleba; (**E**,**F**) medullary excipule with pseudoparenchymatous structure, formed by subglobose or slightly elongated sclerenchyma cells; (**G**,**H**) ascus with three and four ascospores; (**I**) ascospores. Scale bars: (**C**) = 2 mm; (**D**) = 40 µm; (**E**,**F**) = 20 µm; (**G**,**H**) = 20 µm; (**I**) = 5 µm. Photos: A. Paz. Images (**A**,**B**) taken from dry material; (**C**–**I**) taken from rehydrated material.

**Figure 9:**
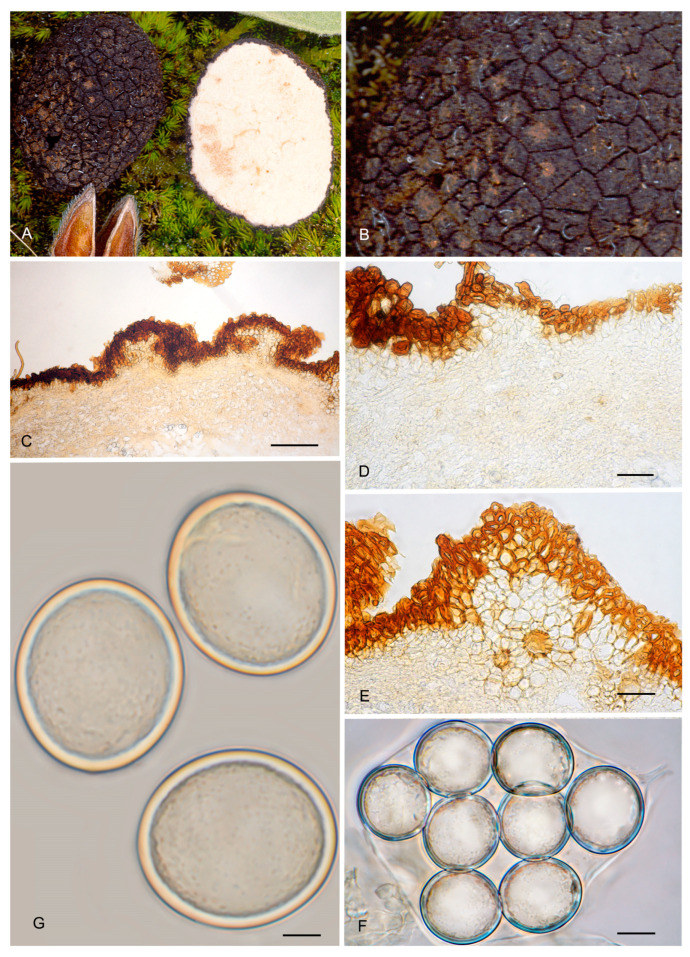
*Picoa cordubensis* BCN-myc 3582 (holotype): (**A**) ascomata; (**B**) detail of surface; (**C**) section of peridium and gleba; (**D**,**E**) medullary excipulum with pseudoparenchymatous structure, made of subglobose or slightly elongated sclerenchymatous cells and with the presence of large globose cells; (**F**) ascus with eight ascospores; (**G**) ascospores. Scale bars: (**C**) = 2 mm; (**D**,**E**) = 40 µm; (**F**) = 10 µm; (**G**) = 5 µm. Photos: (**A**,**B**) = J. Gómez; (**C**–**G**) = A. Paz. All images were taken from fresh material.

**Figure 10:**
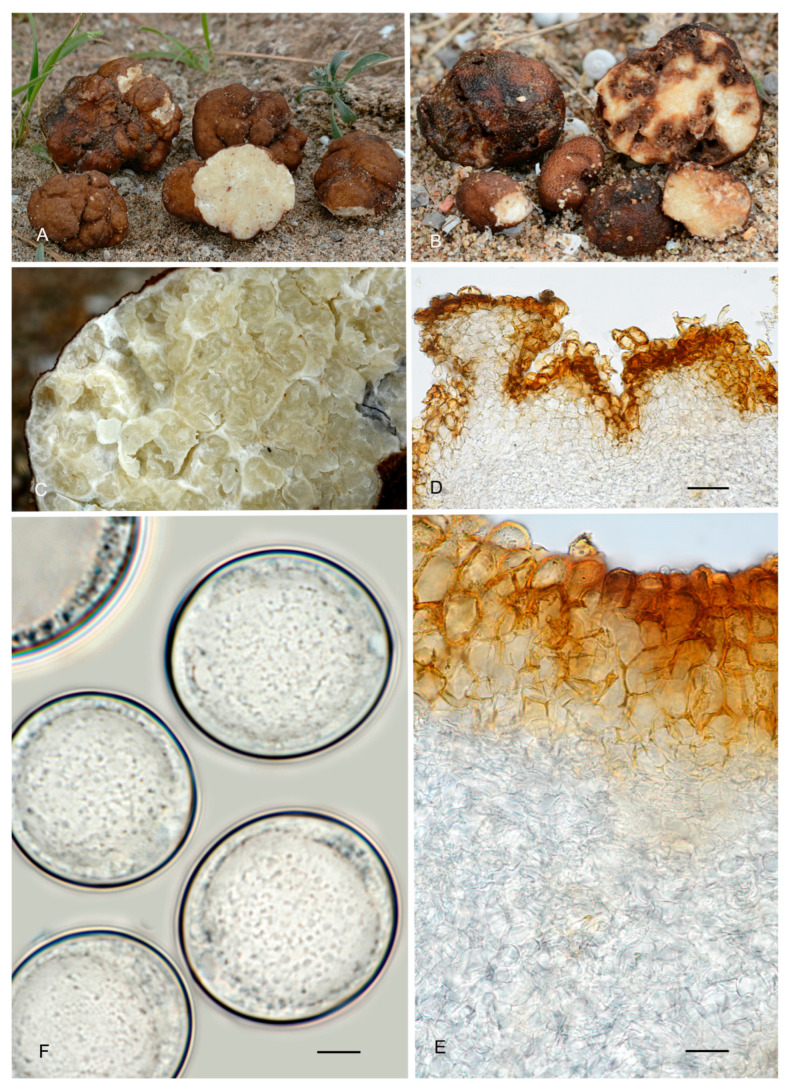
***Picoa llobregatina*** BCN-myc 3586 (holotype): (**A**) ascomata; (**B**) ascomata BCN-myc 3587; (**C**) detail of gleba; (**D**) section hyphae peridium and gleba; (**E**) medullary excipulum with pseudoparenchymatous structure, made of parallelepiped cells; (**F**) ascospores. Scale bars: (**D**) = 40 µm; (**E**) = 20 µm; (**F**) = 5 µm. Photos: (**A**,**C**–**F**) = A. Paz; (**B**) = B. L. Sánchez. All images were taken from fresh material.

**Figure 11:**
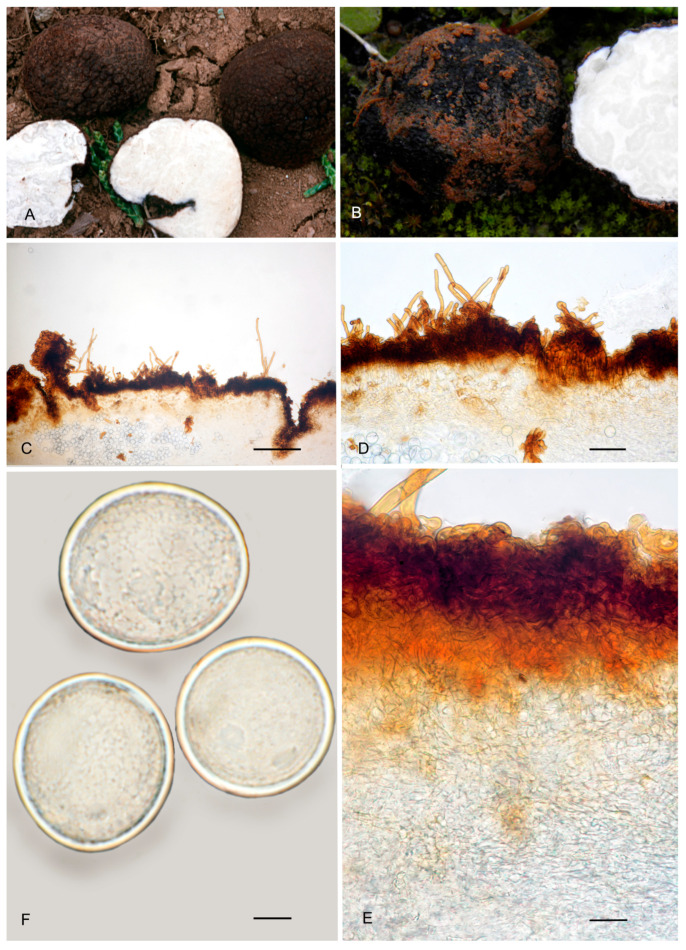
*Picoa pubescens*: (**A**) ascomata (BCN-myc 3591, holotype); (**B**) surface detail (VK5561); (**C**–**F**) BCN-myc 3591 holotype: (**C**) section of peridium and gleba; (**D**,**E**) medullary excipulum with pseudoparenchymatous structure, made of cells that are parallelepiped, arranged in vertical parallel; (**F**) ascospores. Scale bars: (**C**) = 2 mm; (**D**) = 40 µm; (**E**) = 20 µm; (**F**) = 5 µm. Photos: (**A**) = A. Paz; (**B**) = V. Kaounas; (**C**–**F**) = A. Paz. All images were taken from fresh material.

3. ***Picoa juniperi*** Vittad. *Monogr. Tuberac*.: 55. 1831.—Table II, Figure VIII. MB 227320. Figure 3, Figure 4, Figure 5 and Figure 6.
  Original description [1]: “*Uterus rotundus vel leviter compressus, extus niger, minute verrucosus, verrucis adpressis parum elevatis. Caro mollis, exsucca, granulosa, subfriabilis. Venae pallidae, irregulares, interruptae, emarginatae, confusae, e variis peridii punctis in carnem dispersae. Odor ingratus. Fungus magnitudine pisi vel nucis avellanae, raro juglandis; regularissimus, cortice tenui vestitus, fovea seu rima basilari destitutus. Caro (sporangifera) albida, immutabilis; venae primo albidae, parum visibiles, demum pallidae. Vulgo Morandini, Trifole del Ginepro. In sylvis collium et montium transpadanorum, circa Juniperos praesertim obvius. Autumno recedente et ineunte hieme cum Tuberibus effoditur, ipsisque commixtus in foro frequentissime exstat. Caro ejus insipida, foetida, nauseosa nec esculenta”*.
  *Type*: Table II, Figure VIII in Vittadini [1] (lectotype here designated MBT 10027165, Figure 3A); BCN-myc 3617 (epitype here designated MBT 10027166). See *Specimens examined*.
  Ascomata form small, globose stereothecia, 21–28 mm diam., dark brown when young, becoming black when mature, with abundant mycelial hairs in youth which disappear when mature. Peridium (surface) with compound warts measuring 290–360 µm (height) × 673–802.5 µm (base), very irregular in shape, often joining together, with a crater-shaped center and slightly pronounced vertical edges of 3–6 µm. Medullary excipulum (peridium section) arranged in three layers: (1) outer layer 57–130.5 µm thick on average, textura angularis, formed by 3–5 rows of cells at the apices of the warts and 2–3 at the bases, with a pseudoparenchymatous structure, made of subglobose to elongated sclerenchymatous cells, 20.5–26.5 × 22.5–33 µm, Q = 0.93, very thick-walled, up to 10.9 µm high, highly pigmented dark brown, measuring on average 28 × 13.5 µm, Q = 2.06, and interspersed with bases of some cylindrical mycelial hairs; (2) intermediate layer quite thick, measuring on average 214 (high at the wart section)–60.3 (high at the base of the warts) µm, textura angularis, with a pseudoparenchymatous structure, made of hyaline, sclerenchymatous, slightly elongated polyhedric cells, 15–22.5 × 24–32 µm, Q = 0.67, and thin walls up to 3.6 µm thick; (3) inner layer (transition layer toward the gleba) thick, 126–250 µm thick, textura angularis, made of increasingly smaller polyhedric cells, 8.5–14.5 × 8–21 µm Q = 0.95, interspersed with elongated, interwoven, septate and bifurcate cells of the glebal tissue. Gleba formed by (1) sterile zones with an intricate texture, composed of elongated, bifurcate hyphae, with septa measuring an average of 9.5 × 4 µm and (2) irregularly arranged fertile cells, formed by quickly disintegrating asci. Asci 158.5–*162*–167 × 69.5–*70.5*–72 µm, prototunicate, subglobose, with a peduncle 40–55 µm long (included in the ascus measurements), thin-walled, containing 6–8 ascospores. Ascospores broadly ellipsoidal, 27–*28*–29 × 25–*25.5*–26.5 µm, Q = 1.06–1.15, ornamented with thick, isolated granules and small roughness (when fully mature).
  *Ecology and Distribution*: Collected in Italy and Spain, under *Quercus pubescens*, *Pinus* sp., and *Juniperus communis*, in sandy soil (southern slope), and under *Quercus ilex*, in clay and calcareous soil.
  *Specimens examined*: **France**, 1873, *Tulasne*, PC 0022113. **Italy**, Emilia-Romagna Bologna, Guzzano Pianoro, mixed forest of *Quercus pubescens, Pinus* sp., *Juniperus communis*, sandy soil, south slope, 10 December 1998, *A. Zambonelli* & *M. Morara* AZ1404 (BCN-myc 3617* epitype; duplicate in pers. herb. A. Paz as IC10129801). **Spain**, Burgos, Covarrubias, under *Quercus ilex*, 6 September 2011*, L. Barrio* (BCN-myc IC06091101*); Catalonia, Girona, Maçanet de Cabrenys, Bac Grillera, La Fillola, 750 m, *Quercus ilex*, calcareous clay soil, 28 April 2001, *J-M Vidal* (JMV20010428-3*).
  *Notes*: The main diagnostic features mentioned by Vittadini [1] in his original description were that this species was collected under *Juniperus* sp. in autumn and winter along with other edible truffles, its small size (no more than a hazelnut), and a nauseating smell. Morphologically, *P. juniperi* is characterized also by having (1) sparse mycelial hairs at maturity; (2) surface with irregular compound warts, close together and not very pronounced (with 3–6 edges); (3) outer layer of the peridium thick (2–5 rows of cells), pseudoparenchymatous, sclerenchymatous cells with a thick wall not exceeding 10 µm; (4) internal layer very thick, pseudoparenchymatous, composed of polyhedric cells interspersed with elongated forked hyphae; (5) ascospores broadly ellipsoidal, on average 28 × 25.5 µm, Q = 1.11, ornamented with thick, isolated granules and slight roughness; (6) preference for calcareous soils; (7) probably associated with *Quercus*; (8) fruiting in late summer and autumn. The most closely related species is *P. macrospora*, which has permanent mycelial hairs at maturity, surface with very pronounced pyramidal compound warts, a thinner internal layer of the peridium formed by an intricate plectenchymatous structure, and larger ascospores, 29–*31*–31.5 × 26.5–*28*–29.5 µm, Qm = 1.10, ornamented with very fine, granules, which join together at the bases, giving the impression of forming semi-reticles.

4. ***Picoa macrospora*** A. Paz, R. Martínez & C. Lavoise, *sp. nov*. MB 859753. Figure 3, Figure 4 and Figure 5 and Figure 7.
  *Etymology*: From the ancient Greek adjective μακρός (*makrós*), meaning large, and *spora*, meaning seed, in relation to the size of ascospores, the largest of the known species.
  *Type*: **Spain**, La Rioja, Ortigosa de Cameros, Sierra de Cebollera Natural Park, under *Quercus ilex*, 13 December 2012, *R. Martinez* 2159 (BCN-myc 3579 holotype, IC13121332 isotype).
  Ascomata form globose stereothecia, 25–38 mm diam., black, with a few mycelial hairs. Peridium (surface) with very large compound pyramidal warts, measuring 162–417.5 µm (height) × 159.5–761.5 µm (base) and remarkable vertical edges of 3–6 µm. Medullary excipulum (peridium section) arranged in three layers: (1) outer layer about 59–146 µm thick, formed by 3–6 rows of cells at the apices of warts and 2–3 at the bases, textura angularis, with a pseudoparenchymatous structure made of subglobose or slightly elongated sclerenchymatous cells, 11–34 × 11.5–26 µm, Q = 1.11, thick-walled (up to 11 µm), with dark reddish brown to almost black walls. This layer contains also some isolated bases of cylindrical mycelial hairs, measuring on average 22.5 × 11 µm, Q = 2.05; (2) intermediate layer 109–178.48 µm thick, textura angularis, with a pseudoparenchymatous structure made of globose to polyhedric hyaline cells, 9.5–28.5 µm × 7.5–29 µm, Q = 1.19, interspersed with more elongated cells; (3) internal layer (transition toward the gleba) 92.5–146 µm thick, textura intrincata, arranged as an intricate plectenchymatous structure made of increasingly smaller and elongated cells, 13–28.5 × 6–13.5 µm, Q = 0.56. Gleba with irregularly arranged fertile cells formed by quickly disintegrating asci. Asci 163.5–*165.5*–168 × 72–*72.5*–73.5 µm, inamyloid, prototunicate, subglobose, with a long peduncle, thin-walled, and containing 6–8 ascospores. The sterile zones of the gleba have an intricate texture formed by long and bifurcate hyphae, with septa measuring 11 × 4 µm on average. Ascospores broadly ellipsoidal, 29–*31*–31.5 × 26.5–*28*–29.5 µm, Q = 1.09–1.15, with walls in gold-brown tones, ornamented with tiny granules that join together at their bases, giving the impression of forming semi-reticles.
  *Ecology and Distribution*: Autumnal species, collected in northern Italy and central Spain, usually under *Quercus ilex*, sometimes with the presence of *Juniperus*, showing preference for calcareous soils.
  *Additional specimens examined*: **Italy**, Piedmont, Cuneo, Alba, under *Quercus ilex,* 5 November 2008, *A. Paz* (IC05110807). **Spain**, Castilla y León, Zamora, Peleas, *Q. ilex*, 1 December 2013, *J. Cabero* JC131201NR (BCN-Myc IC15091531); ibid. (BCN-myc 3581*, IC15091532); La Rioja, Almarza de Cameros, Ribavellosa farm, under *Q. faginea*, *Q. ilex* and *Juniperus communis*, 11 November 2012, *R. Martinez* 2179 (BCN-myc 3580*-IC11111234).
  *Notes*: *Picoa macrospora* is characterized by (1) permanent mycelial hairs at maturity; (2) surface with very pronounced compound pyramidal warts (with 2–3 edges); (3) thick outer layer of the peridium formed by 3–6 rows of pseudoparenchymatous and sclerenchymatous subglobose to slightly elongated cells, with thick walls (up to 11 µm); (4) thinner internal layer of the peridium formed by an intricate plectenchymatous structure of elongated cells; (5) ascospores broadly ellipsoidal, ornamented with very fine granules, which join together, giving the impression of forming semi-reticles; (6) preference for calcareous soils; (7) probably associated with *Quercus*; (8) fruiting in late autumn. The most closely related species is *P. juniperi* (see above).

5. ***Picoa*** **sect. *Lefebvreorum*** P. Alvarado, A. Paz & Van Vooren, *sect. nov*. MB 859772. Figure 3, Figure 4 and Figure 5 and Figure 8, Figure 9, Figure 10 and Figure 11.
  *Type*: *Picoa lefebvrei* (Pat.) Maire.
  *Etymology*: Named after the type, *P. lefebvrei.*
  The section *Lefebvreorum* is characterized by (1) fawn-reddish brown to black ascomata, with a warted or granulose surface, (2) outer peridium layer formed by parallelepipedic sclerenchymatous cells, (3) broadly ellipsoidal ascospores, measuring up to 27 µm long, ornamented with fine granules that join together to form ridges or semi-reticles, (4) ecologically linked to *Cistaceae* (*Helianthemum*, *Cistus*, *Fumana*) sometimes with the presence of *Quercus*, *Pinus* and/or *Juniperus*, in sandy soils. The section is composed of *P. cordubensis*, *P. lefebvrei*, *P. llobregatina*, and *P. pubescens*, but it probably includes multiple taxa that need to be reviewed with additional data.

**Key to the sections of *Picoa* sect. *Lefebvreorum***

1.Associated with perennial *Helianthemum.*21.Associated with *Cistus* or *Fumana.*32.Ascomata fawn-reddish brown, surface showing minute isolated granules with a halo at their base, ascospores 24–*25*–26 × 20.5–*21*–22.5 µm.
*P. llobregatina.*
2.Ascomata reddish brown when young and grayish brown when mature, surface with simple, small, flat warts, ascospores 26.5–*27*–27.5 × 22–*22.5*–23 µm.*P.* grex *lefebvrei.*3.Ascomata with abundant mycelial hairs when young, then disappearing, internal layer of the peridium pseudoparenchymatous, made of increasingly smaller, elongated puzzle-like cells, ascospores 25–*26.5*–27 × 22.5–*23.5*–30 µm.
*P. cordubensis.*
3.Ascomata dark reddish brown when young, black when mature, with abundant and persistent mycelial hairs, internal layer of the peridium plectenchymatous, intricate, ascospores 24.5–*25*–25.5 × 22–*22.5*–23 µm.
*P. pubescens.*

**Figure 12:**
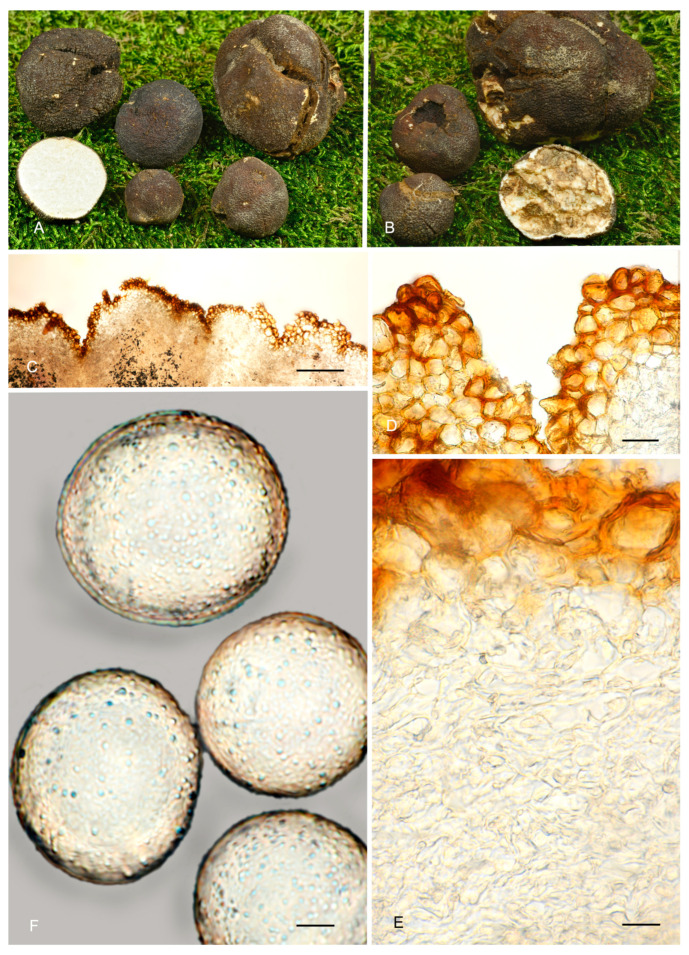
*Picoa communis* BCN-myc 3592 (holotype): (**A**) ascomata; (**B**) detail of peridium and gleba section; (**C**) section of peridium and gleba; (**D**,**E**) medullary excipulum with pseudoparenchymatous structure, made of subglobose to polyhedric sclerenchymatous cells; (**F**) ascospores. Scale bars: (**C**) = 2 mm; (**D**) = 40 µm; (**E**) = 20 µm; (**F**) = 5 µm. Photos: A. Paz. All images were taken from fresh material.

**Figure 13:**
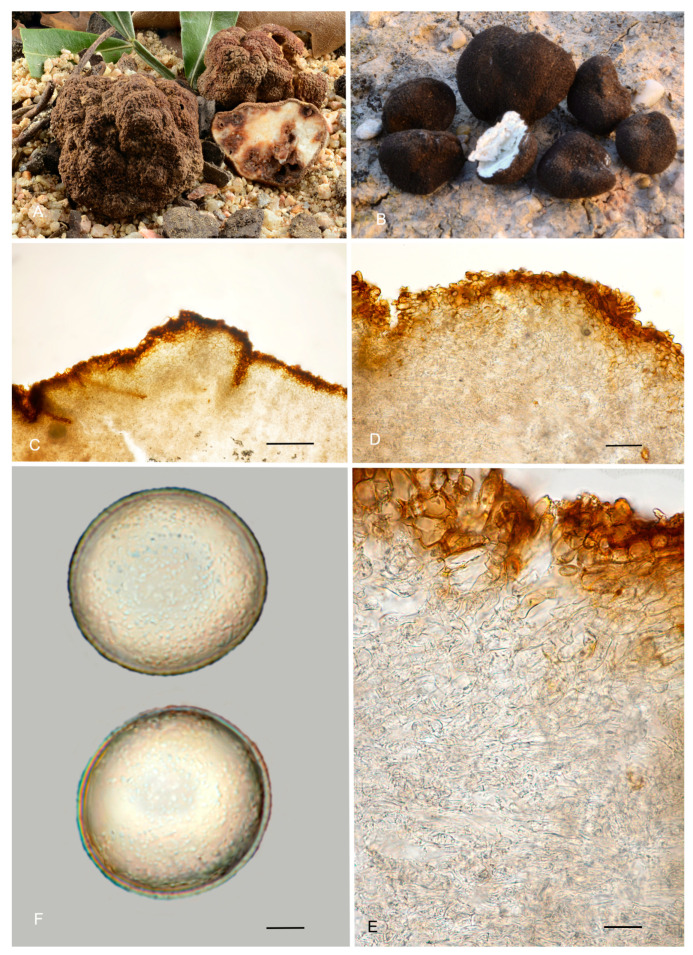
*Picoa fajardoi* BCN-myc 3595 (holotype): (**A**) ascomata; (**B**) detail of peridium and gleba section; (**C**) prominent pyramidal warts, shaped like a double-inverted triangle; (**D**,**E**) medullary excipulum with pseudoparenchymatous structure; (**F**) ascospores. Scale bars: (**C**) = 2 mm; (**D**) = 40 µm; (**E**) = 20 µm; (**F**) = 5 µm. Photos: (**A**,**C**–**F**) = A. Paz; (**B**) = E.-J. Salvador. All images were taken from fresh material.

**Figure 14:**
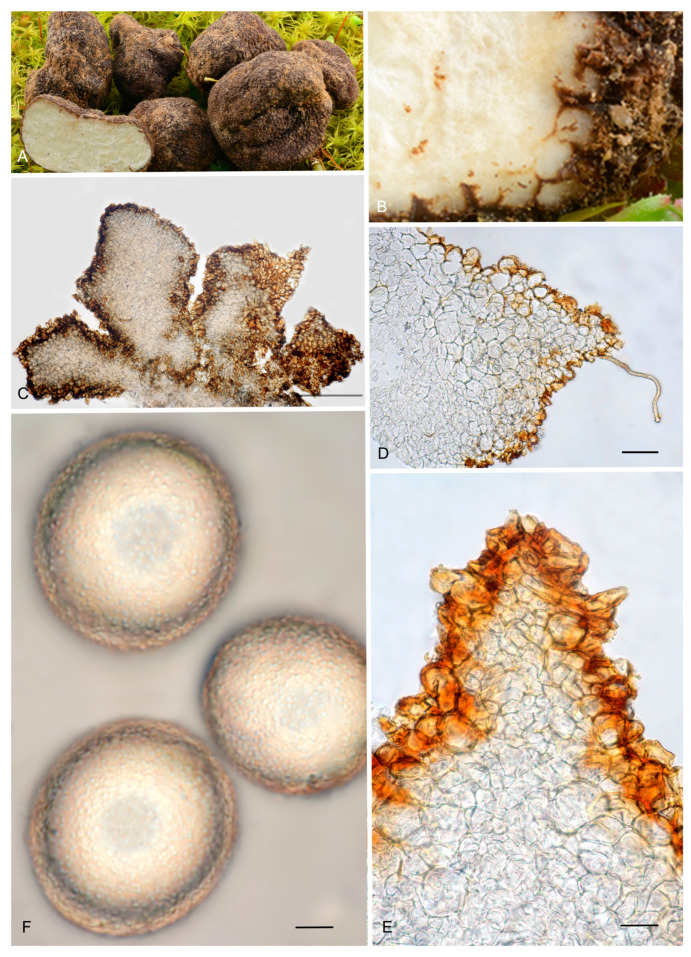
*Picoa montecchii* BCN-myc 3598 (holotype): (**A**) ascomata; (**B**) detail of peridium and gleba section; (**C**) prominent pyramidal warts, shaped like a double-inverted triangle; (**D**,**E**) medullary excipulum with pseudoparenchymatous structure; (**F**) ascospores. Scale bars: (**C**) = 2 mm; (**D**) = 40 µm; (**E**) = 20 µm; (**F**) = 5 µm. Photos: A. Paz. All images were taken from fresh material.

**Figure 15:**
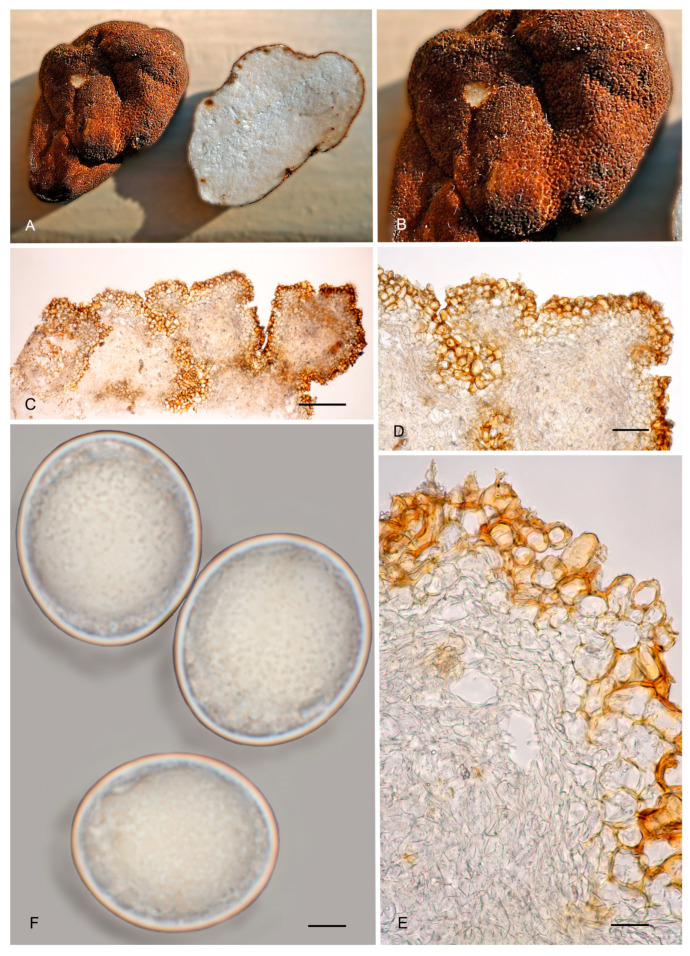
*Picoa morarae* BCN-myc 3600 (holotype): (**A**) ascomata; (**B**) surface detail; (**C**) section of peridium and gleba; (**D**,**E**) medullary excipulum with pseudoparenchymatous structure, made of globose sclerenchymatous cells; (**F**) ascospores. Scale bars: (**C**) = 2 mm; (**D**) = 40 µm; (**E**) = 20 µm; (**F**) = 5 µm. Photos: (**A**,**B**) = M. Morara (from fresh material); (**C**–**F**) = A. Paz (rehydrated material).

**Figure 16:**
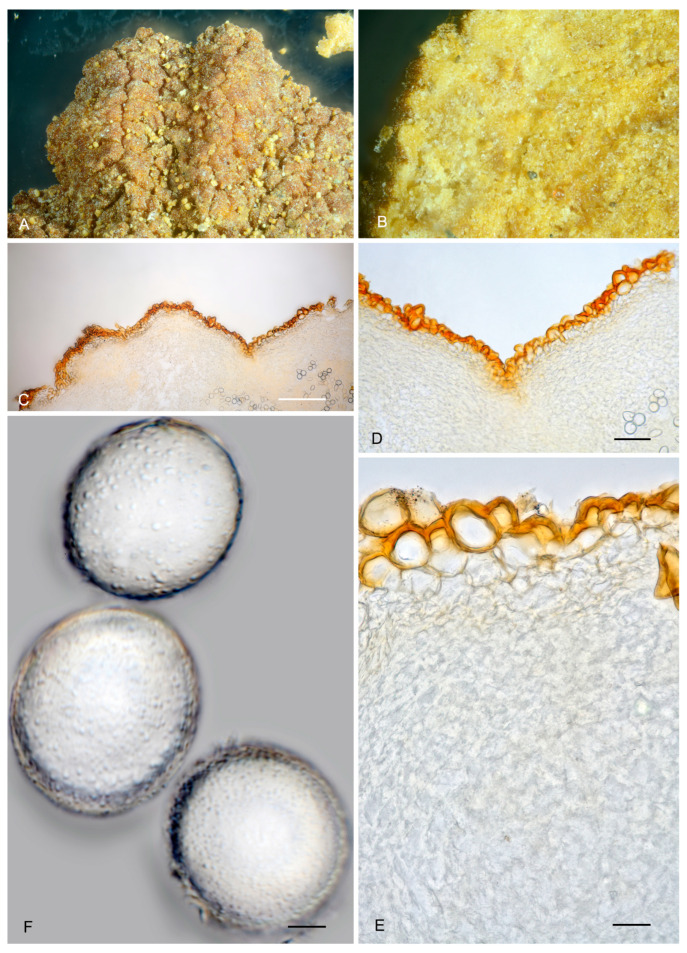
*Picoa pacionii* BCN-myc 3601 (holotype): (**A**) ascomata; (**B**) surface detail; (**C**) section of peridium and gleba; (**D**,**E**) medullary excipulum with pseudoparenchymatous structure, made of very small subglobose sclerenchymatous cells; (**F**) ascospores. Scale bars: (**C**) = 2 mm; (**D**) = 40 µm; (**E**) = 20 µm; (**F**) = 5 µm. Photos: A. Paz. Images (**A**,**B**) taken from dry material; (**C**–**F**) taken from rehydrated mounts.

**Figure 17:**
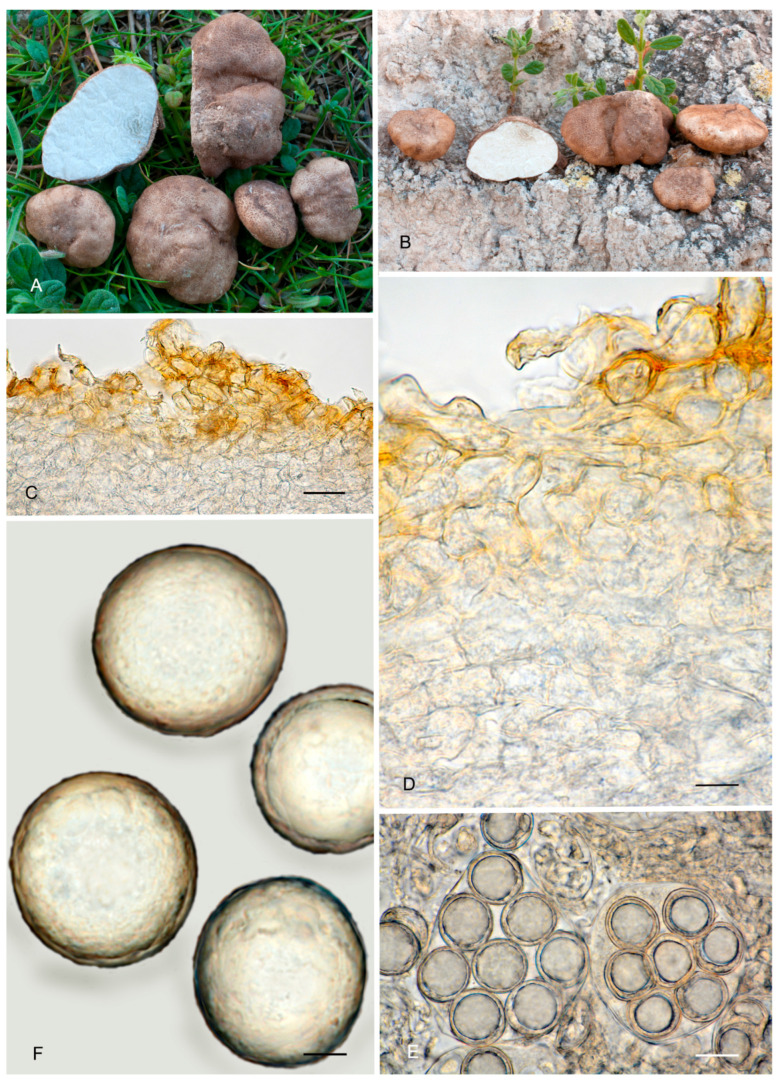
*Picoa papillata* BCN-myc 3602 (holotype): (**A**,**B**) ascomata; (**C**,**D**) medullary excipulum with pseudoparenchymatous structure, made of very large subglobose sclerenchymatous cells; (**E**) ascus with seven to eight ascospores; (**F**) ascospores. Scale bars: (**C**) = 40 µm; (**D**) = 20 µm; (**E**) = 10 µm; (**F**) = 5 µm. Photos: (**A**,**B**) = J. A. Martínez; (**C**–**F**). A. Paz. All images were taken from fresh material.

**Figure 18:**
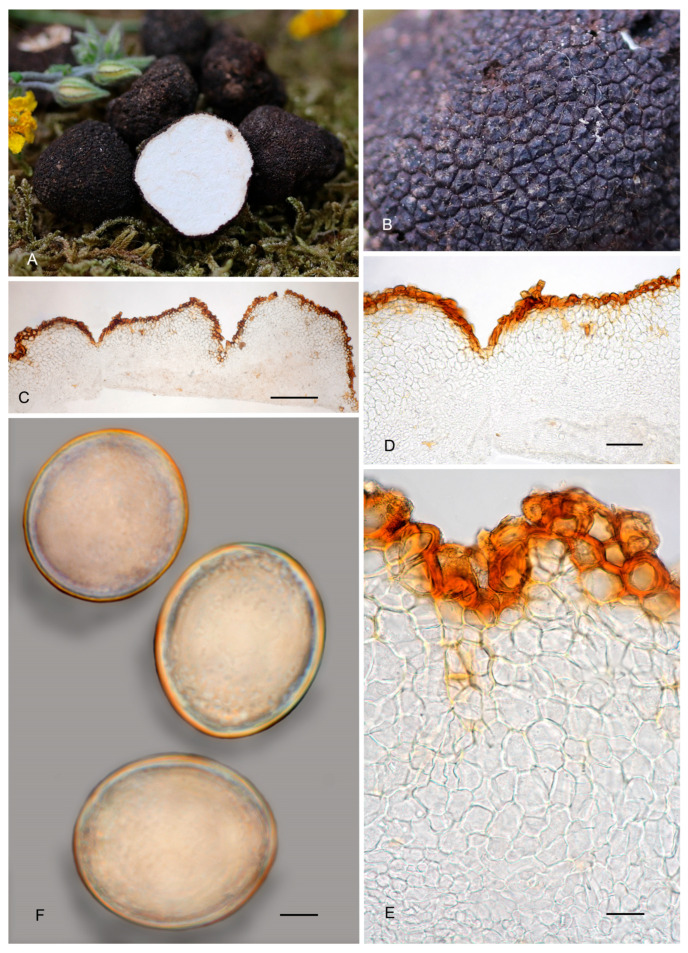
*Picoa pontica* BCN-myc 3603 (holotype): (**A**) ascomata; (**B**) Surface detail; (**C**) section of peridium and gleba; (**D**,**E**) medullary excipulum with pseudoparenchymatous structure, made of subglobose or slightly elongated sclerenchymatous cells; (**F**) ascospores. Scale bars: (**C**) = 2 mm; (**D**) = 40 µm; (**E**) = 20 µm; (**F**) = 5 µm. Photos: (**A**,**B**) = M. Slavova (from fresh material); (**C**–**F**) = A. Paz (from rehydrated material).

**Figure 19:**
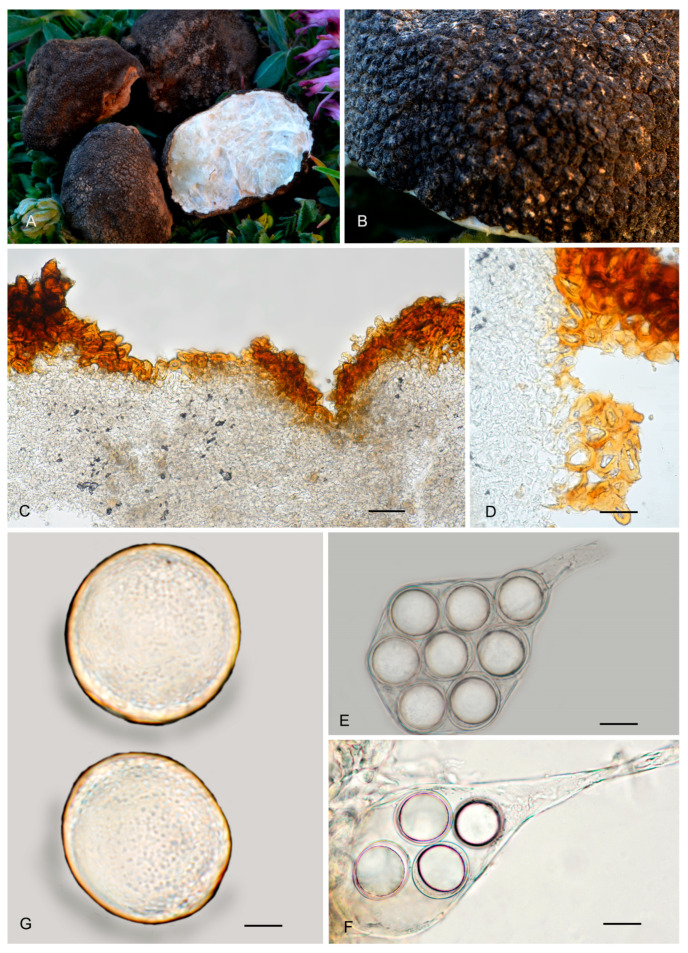
*Picoa rodensis* BCN-myc 3606 (holotype): (**A**) ascomata; (**B**) surface detail; (**C**) section of peridium and gleba; (**D**) medullary excipulum with pseudoparenchymatous structure, made of subglobose or slightly elongated, very irregular in size; (**E**,**F**) ascus with four to eight ascospores; (**G**) ascospores. Scale bars: (**C**) = 40 µm; (**D**) = 20 µm; (**E**,**F**) = 10 µm; (**G**) = 5 µm. Photos: (**A**,**B**) = E. J. Salvador-Fernández; (**C**–**G**) = A. Paz. All images were taken from fresh material.

**Figure 20:**
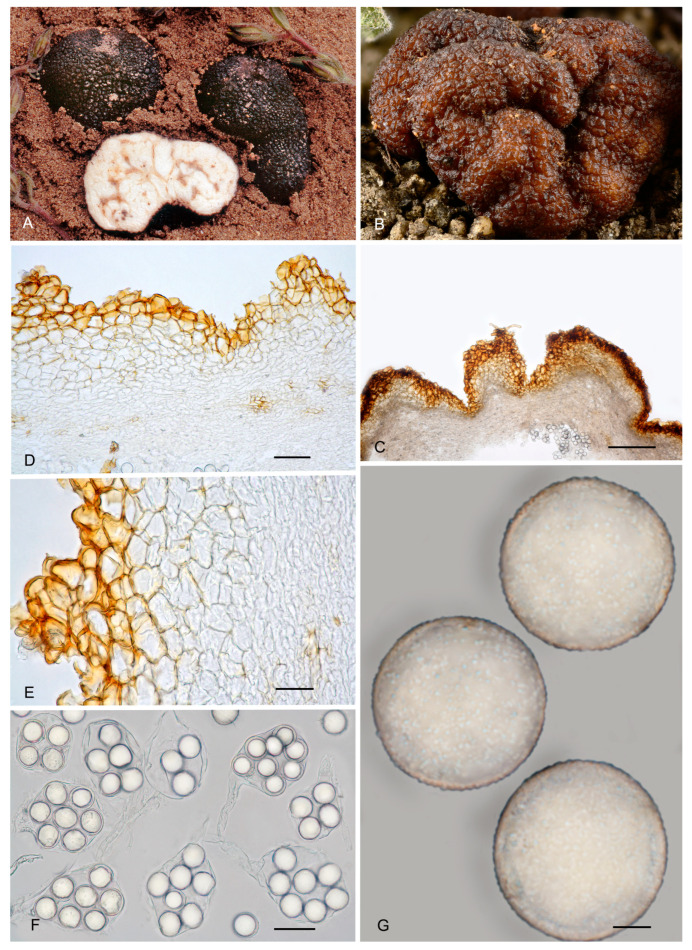
*Picoa sphaerospora* BCN-myc 3608 (holotype): (**A**) ascomata; (**B**) surface detail; (**C**) section of peridium and gleba; (**D**,**E**) medullary excipulum with pseudoparenchymatous structure, made of subglobose to polyhedric sclerenchymatous cells; (**F**) ascus with 3–8 ascospores; (**G**) ascospores. Scale bars: (**C**) = 2 mm; (**D**) = 40 µm; (**E**,**F**) = 20 µm; (**G**) = 5 µm. Photos: (**A**) = J. Gómez; (**B**–**G**) = A. Paz. All images were taken from fresh material.

**Figure 21:**
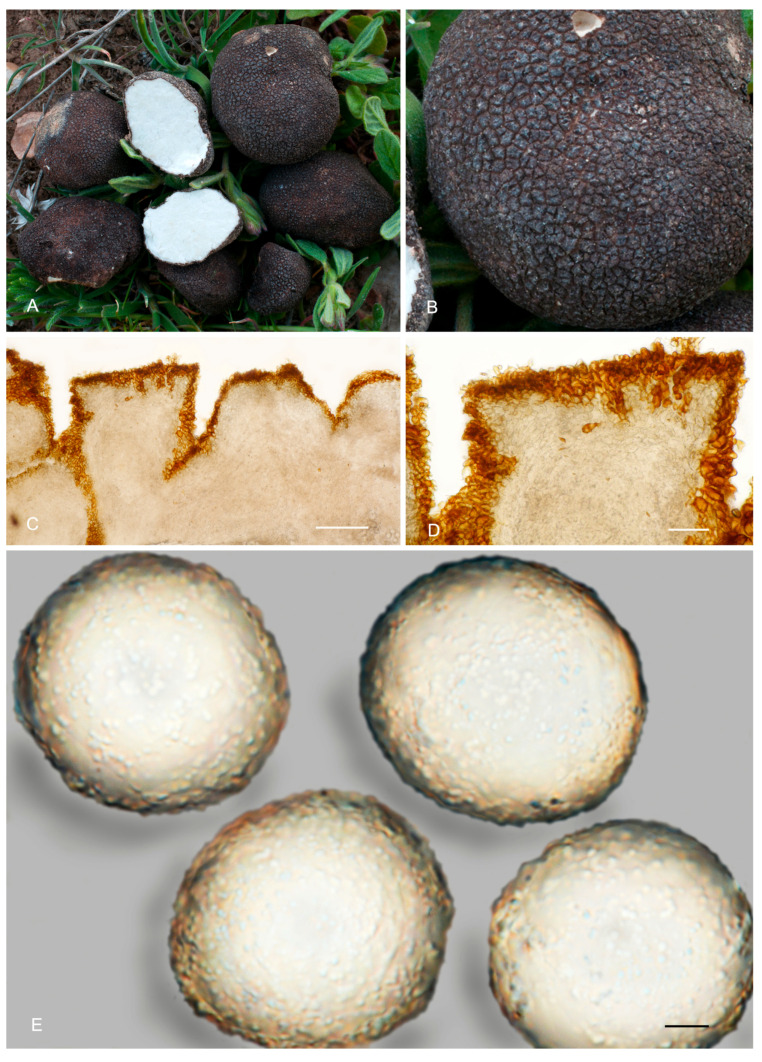
*Picoa truncata* BCN-myc 3610 (holotype): (**A**) ascomata; (**B**) surface detail; (**C**) section of peridium and gleba; (**D**) medullary excipulum with pseudoparenchymatous structure, made of subglobose sclerenchymatous cells; (**E**) ascospores. Scale bars: (**C**) = 2 mm; (**D**) = 40 µm; (**E**) = 5 µm. Photos: (**A**,**B**) = J.–A. Martínez; (**C**–**E**) = A. Paz. All images were taken from fresh material.

**Figure 22:**
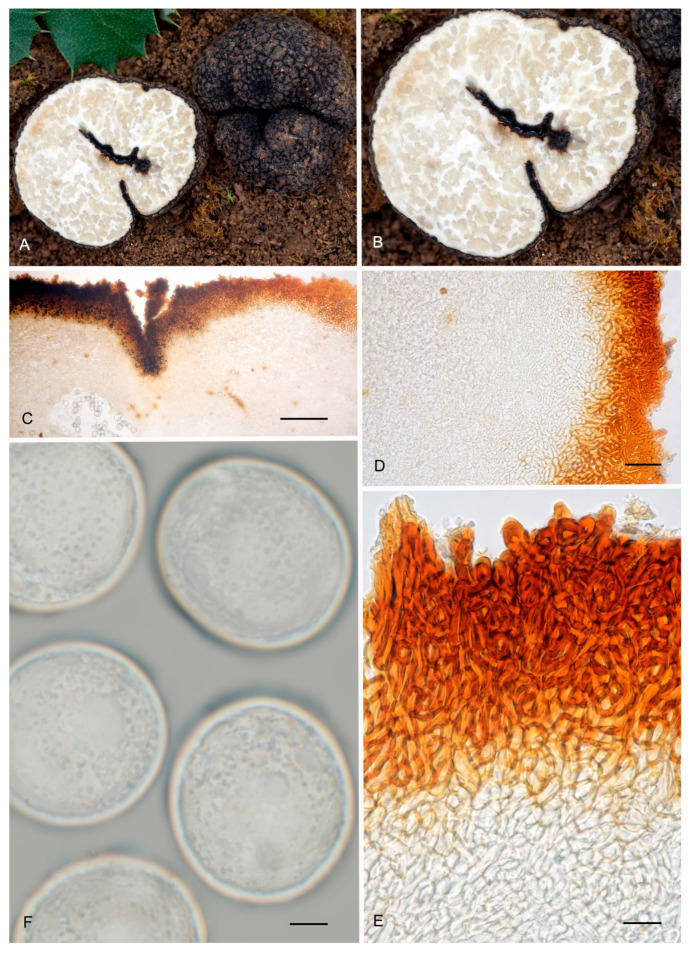
*Picoa vazqueziae* BCN-myc 3611 (holotype): (**A**) ascomata; (**B**) detail of gleba; (**C**) section of peridium and gleba; (**D**) medullary excipulum with pseudoparenchymatous structure, made of small, parallelepiped, elongated cells, vertically parallel; (**E**) ascospores. Scale bars: (**C**) = 2 mm; (**D**) = 40 µm; (**E**) = 20 µm; (**F**) = 5 µm. Photos: (**A**,**B**) = J. Cabero (from fresh material); (**C**–**E**) = A. Paz (from rehydrated material).

6. ***Picoa lefebvrei*** (Pat.) Maire, *Annls Mycol*. 4: 332. 1906. MB 456009. Figure 3, Figure 4 and Figure 5 and Figure 8.
  ≡ *Phaeangium lefebvrei* Pat., *J. Bot*. (Morot) 8: 155. 1894. MB 120769.
  **=*** Terfezia schweinfurthii* Henn., Beibl. Hedwigia 40: 100. 1901. MB 139798.
  *Typus*: FH 301557 (holotype of *Phaeangium lefebvrei*).
  Ascomata form globose stereothecia, 21–28 mm diam., reddish brown when young, becoming grayish brown when mature, showing mycelial hairs (even in maturity) that raise from the intermediate peridium layer. Peridium (surface) crackled when fresh, formed by large flat warts, 154.5–269 µm (height) × 279.5–585 µm (base), but granulose in exsiccata. Medullary excipulum (peridium section) arranged in three layers: (1) a thin outer layer measuring about 76.5–138.5 µm thick, composed of 3–4 rows of cells at the apices and 2–3 rows at the bases, textura angularis, forming a pseudoparenchymatous structure made of very small, subglobose sclerenchymatous cells, 8–17.5 × 7–20.5 µm, Q = 1.08, with walls up to 4.7 µm thick, reddish brown pigmented; this layer has abundant bases of mycelial hairs (raising from the intermediate layer), cylindrical in shape, and slightly widened at the bases, measuring on average 19 × 12 µm; (2) an intermediate layer about 435–132.5 µm thick, textura angularis, with a pseudoparenchymatous structure made of sclerenchymatous, subglobose cells, very irregular in size, 10.5–20 × 11.5–21.5 µm, Q = 0.96, with thin walls up to 2 µm thick; this layer has elongated cylindrical cells of the hairs of the outer layer; (3) internal layer (transition toward the gleba) 106–190.5 µm thick, with a plectenchymatous structure, made of elongated cells arranged horizontally, 13–26 × 8.5–13 µm, Q = 0.58, and merged with glebal tissue of interwoven hyphae. Gleba with irregularly arranged fertile cells, which acquire a light brown tone. Asci 126.5–*128*–130 × 53–*53.5*–54.5 µm, soon deliquescing, inamyloid, prototunicate, subglobose to broadly ellipsoidal, with a peduncle 30–45 µm long (included in the ascus measurements), with thin walls, containing 3–8 ascospores. The sterile zones of the gleba have an intricate texture, made of elongated bifurcate hyphae, with septa 10 × 3 µm on average. Ascospores ellipsoidal, 26.5–*27*–27.5 × 22–*22.5*–23 µm, Q = 1.16–1.24, ornamented with very fine and not very pronounced granules, which join together forming small irregular ridges or semi-reticles.
  *Ecology and Distribution*: Species collected so far on African continent: Algeria, Egypt, Canary Islands (Spain), and Tunisia under *Helianthemum*, in semi-arid, neutral, sandy soils, fruiting from late winter to spring.
  *Specimens examined*: **Algeria**, Bechar, Beni Ounif (BMBO20*); ibid. (PON1 BMBO21*, PON3 BMBO23*, PON4 BMBO24*, PRBe1 BMBT28*, PRBe2 BMBT29*, PRBe3 BMBT30*, PW1 BMBO18*, PW2 BMBO19*); Hauts-Plateaux à Chellala, March 1922, *Maire* (M 157945); near Biskra, 1901 (S F8693, syntype of *Terfezia schweinfurthii*); Tabelbala (BMBT27*, BMBT25*, BMBT26*). **Egypt**, Masrab El Khanyha, 17 February 1993, *G. Pacioni* & *El Kholy* (AQUI7043*); ibid. (AQUI7046*). **Spain**, Canary Islands, Fuerteventura, Betancuria, 412 m, 13 March 2010, *M-A Ribes* & *V. Escobio* (MAR13031063a* brown); ibid. (MAR13031063b* black); ibid. SMGC2015021803*); Canary Islands, Gran Canaria, Las Palmas de Gran Canaria, El Lasso, between the barranco “del Cortijo” or “de los Toledo” and the “Lomo del Sabinal”, sandy detritic soil with *Euphorbia balsamifera*, *Helianthemum canariense*, *Senecio kleinia*, *Artemisia reptans*, *Rubia fruticosa*, *Ceropegia fusca*, *Aeonium percarneum,* and several Poaceae, 180 m asl, 18 February 2015, *Y. Toledo & J. Santiago* (SMGC2015031408); Canary Islands, Gran Canaria, Molinos de Piso Firme, Agaete, abandoned crops with *Helianthemum canariense* and *Euphorbia regis-jubae*, 195 m asl, 9 February 2019, *C.C. Rodríguez-Cabrera* (SMGC2019020901). **Tunisia**, between Ras-el-Oued and El Hamdon, 1894, *Lefebvre* (FH 301557, under *Phaeangium lefebvrei,* holotype). Mahdia Central, area with a semi-arid climate, April 2008 (TUNISIA—4 IRA-MBA SBc*); Medenine, South-East, area with an arid climate, near *Helianthemum lippii* var. *sessiliflorum*, 12 March 2015 (TUNISIA—1 IRA-MBA Sba*).
  *Notes*: *Picoa lefebvrei* is characterized by having (1) reddish brown ascomata when young and grayish brown when mature, with permanent mycelial hairs (very few and isolated); (2) crackled surface with simple small flat warts; (3) a thin outer peridium layer (2–4 rows of cells), pseudoparenchymatous, made of very small subglobose sclerenchymatous cells (13 × 12.5 µm) with thin walls up to 4.7 µm thick; (4) a plectenchymatous internal peridium layer, made of elongate hyphae arranged horizontally; (5) ellipsoidal ascospores ornamented with very fine granules, which join together forming small irregular ridges or semi-reticles; (6) preference for neutral soils; (7) associated with perennial *Helianthemum* species. The most closely related species are *P. cordubensis*, *P. llobregatina,* and *P. pubescens*. *Picoa cordubensis* is distinguished by having dark brown ascomata when young and becoming black when mature; surface with not very pronounced compound pyramidal warts (with 3–5 edges); outer layer of the peridium with cell walls up to 10 µm thick; and an intermediate layer of the peridium with interspersed globose cells. *Picoa llobregatina* is distinguished by a surface showing minute, isolated granules with a halo at their base; outer layer of the peridium made of parallelepipedic cells vertically aligned; internal layer is pseudoparenchymatous, made of puzzle-shaped cells; slightly smaller ascospores of 25 × 21 µm on average, Q = 1.18; occurrence in dunes, in slightly calcareous soils. *Picoa pubescens* has dark reddish brown ascomata when young, becoming black when mature; surface with irregular compound warts and permanent and very abundant mycelial hairs; outer layer of the peridium with parallelepipedic cells vertically aligned, and slightly smaller ascospores, 25 × 22.5 µm on average, Q = 1.11.

7. ***Picoa cordubensis*** A. Paz, J. Gómez, B. Moreno-Arroyo & C. Lavoise, *sp. nov*. MB 859755. Figure 3, Figure 4 and Figure 5 and Figure 9.
  *Etymology*: The epithet refers to Corduba, the Latin name for Córdoba (Spain), the region where this species was first collected.
  *Type*: **Spain**, Andalucia, Córdoba, Priego de Córdoba, Puentes, under *Cistus albidus*, 18 March 1991, *J. Gómez* HB-JG-A40 (BCN-myc 3582 holotype, IC18039109 isotype).
  Ascomata form very small globose stereothecia, 18–26 mm diam., dark brown when young, becoming black when mature, with abundant mycelial hairs in youth. Peridium (surface) with compound pyramidal warts, not very pronounced, and with very large bases, 168–325 µm (height) × 480.5–807.5 µm (base), with a crater-shaped center and 3–5 not very pronounced vertical edges. Medullary excipulum (peridium section) formed by three layers: (1) outer layer about 61.5–95 µm thick, formed by 4–5 rows of cells at the apices and 2–3 at the bases, textura angularis, forming a pseudoparenchymatous structure made of subglobose or elongated and slightly sclerenchymatous cells, 15–43 × 14–36 µm, Q = 1.00, with reddish brown walls, up to 10 µm thick; this layer has abundant bases of mycelial hairs (that sometimes originate in the intermediate layer below), cylindrical in shape but slightly widened at the base, measuring on average 29.5 × 20 µm; (2) intermediate layer measuring about 60–181.5 µm thick, textura angularis, arranged as a pseudoparenchyma, made of hyaline, sclerenchymatous subglobose or slightly elongated cells, 13–39.5 ×11.5–33 µm, Q = 1.14; this layer has also some mycelial hairs with elongated and cylindrical cells measuring on average 40 × 17.5 µm, Q = 2.38; the intermediate layer has also some isolated and large globose cells, measuring 49.5 × 47 µm on average; (3) internal layer (transition toward the gleba) very variable in size, 41.5–188.5 µm thick, textura angularis, with a pseudoparenchymatous structure, made of increasingly smaller elongated puzzle-like cells, 8.5–11 × 13.5–22.5 µm, Q = 0.73; this layer is merged with the underlying glebal tissue composed of interwoven, bifurcate hyphae. Gleba formed by disordered fertile cells among sterile zones, which have an intricate texture, made of elongated and bifurcate hyphae, with septa measuring 8.5 × 3 µm on average. Asci 132–*133.5*–135.5 × 60–*61*–62.5 µm, Q = 2.19, quickly disintegrated, inamyloid, prototunicate, subglobose to broadly ellipsoidal, with a peduncle 35–50 µm long (included in the ascus measurements), with thin walls, containing between 6 and 8 ascospores. Ascospores broadly ellipsoidal, 25–*26.5*–27 × 22.5–*23.5*–30 µm, Q = 1.09–1.13, ornamented with minute granules that join together forming small, very irregular ridges.
  *Ecology and Distribution*: Species collected in southern Spain (Andalusia, Córdoba), under *Cistus* growing in neutral soil, in spring.
  *Additional specimens examined*: **Spain**, Andalusia, Córdoba, Priego de Córdoba, Paraje el Patronati, under *Cistus albidus*, near *Quercus ilex*, 16 January 1993, *B. Moreno–Arroyo & J. Gómez* BM-312 (BCN-myc 3584*); Córdoba, Priego de Córdoba, Paraje Puentes, soil, near *Juniperus oxycedrus* and *Cistus albidus*, 18 March 1991, *B. Moreno-Arroyo & J. Gómez* BM-1059 (BCN-myc 3583*); ibid. 21 March 1993, BM-313*; ibid. 15 May 1993, BM-315 (BCN-myc 3585*).
  *Notes*: *Picoa cordubensis* is characterized by having (1) dark brown ascomata when young, becoming black when mature, with abundant mycelial hairs in youth; (2) surface with not very pronounced compound pyramidal warts (3–5 edges); (3) thick outer layer of the peridium (2–5 rows of cells), pseudoparenchymatous, with subglobose to slightly elongated sclerenchymatous cells, with walls up to 10 µm thick; (4) pseudoparenchymatous intermediate layer of the peridium, composed of subglobose to polyhedrical sclerenchymatous cells with interspersed large globose cells; (5) ascospores widely ellipsoidal, ornamented with minute warts that join together forming irregular small ridges; (6) preference for neutral soils; (7) associated with *Cistus*. The most closely related species are *P. lefebvrei* (see above), *P. llobregatina,* and *P. pubescens*. *Picoa llobregatina* is distinguished by having ascomata fawn-reddish brown, with sparse mycelial hairs, with surface minute isolated granules with a halo at their base; outer layer of the peridium very thin, made of parallelepipedic cells. *Picoa pubescens* has a surface with irregular low compound warts (with 2–3 edges); outer layer of the peridium made of parallelepipedic cells vertically aligned; internal peridium layer plectenchymatous, made of elongated hyphae horizontally arranged.

8. ***Picoa llobregatina*** A. Paz, L. Sánchez & C. Lavoise, *sp. nov*. MB 859756. Figure 3, Figure 4 and Figure 5 and Figure 10.
  *Etymology*: “*llobregatina*” refers to Llobregat Delta Natural Park (Spain), where the holotype of this species was found.
  *Type*: **Spain**, Catalonia, Barcelona, Viladecans, Llobregat Delta Natural Park, Remolar beach, in the fixed dunes with *Pinus pinea*, *Cistus salviifolius*, 16 February 2017, *A. Paz, C. Lavoise* & *L. Sánchez* (BCN-myc 3586 holotype, IC16021701 isotype).
  Ascomata form small globose stereothecia, 21–28 mm diam., fawn-reddish brown, with a few mycelial hairs in youth. Peridium (surface) with isolated, small, irregular granules showing a halo at their base, measuring 147.5–159.5 µm (height) × 222–374 µm (base). Medullary excipulum (peridium section) organized in three layers: (1) outer layer thin and fragile, 32.5–55 µm thick, formed by 2–3 rows of cells at the apices and 1–2 rows at the bases, textura angularis, with a pseudoparenchymatous structure made of parallelepipedic cells, vertically parallel, 18–30.5 × 14–32 µm, Q = 1.23, walls up to 6.5 µm thick, reddish brown pigmented; this layer also has the bases of mycelial hairs, cylindrical, and slightly widened at the bases, measuring on average 23.5 × 20 µm; (2) intermediate layer about 45–109 µm thick, textura angularis, with a pseudoparenchymatous structure made of irregular polygonal sclerenchymatous cells, 15.5–43 × 11–30.5 µm, Q = 1.16; (3) internal layer (transition toward the gleba) very variable in size, 85–150.5 µm thick, textura angularis, with a pseudoparenchymatous structure, made of increasingly smaller puzzle-like cells, 5–10.5 × 10.5–16.5 µm, Q = 0.65; the internal layer is merged in depth with glebal tissue, composed of interwoven long, bifurcate hyphae. Gleba formed by disordered fertile cells that look light brown when mature and sterile zones with an intricate texture, made of long bifurcate hyphae, with septa measuring 10 × 4 µm on average. Asci 146.5–*148.5*–150.5 × 70.5–*72.5*–74.5 µm, quickly evanescent, inamyloid, prototunicate, subglobose to broadly ellipsoidal, with a peduncle 45–55 µm long (included in the ascus measurements), with thin walls; containing between 6 and 8 ascospores. Ascospores broadly ellipsoidal, 24–*25*–26 × 20.5–*21*–22.5 µm, Q = 1.15–1.19, ornamented with minute granules which join together forming very irregular semi-reticles. Smells similar to that of wine.
  *Ecology and Distribution*: Collected only in northeastern Spain, in dunes, in slightly calcareous soil, with the presence of *Pinus pinea* and *Cistus salviifolius*, in February and March.
  *Additional specimens examined*: **Spain**, Catalonia, Barcelona, Viladecans, Remolar beach, in the fixed dunes with *Pinus pinea, Cistus salviifolius*, 15 March 2017, *A. Paz, L. Sánchez* & *C. Lavoise* (BCN-myc 3587*, IC15031701); ibid. 18 February 2018 (BCN-myc 3588, IC18021803); ibid. 12 April 2018 (BCN-myc 3589, IC12041807).
  *Notes*: *Picoa llobregatina* is characterized by having (1) fawn-reddish brown ascomata, with sparse mycelial hairs in youth; (2) surface showing minute isolated granules with a halo at their base; (3) outer layer of the peridium very thin (1–3 rows of cells), pseudoparenchymatous, made of parallelepipedic cells, vertically parallel, with walls up to 6.5 µm; (4) internal layer is pseudoparenchymatous, made of puzzle-shaped cells; (5) ascospores broadly ellipsoidal, ornamented with minute granules that join together forming very irregular semi-reticles; (6) preference for sandy neutral soils; (7) probably associated with *Cistus* or *Pinus*. The most closely related species are *P. cordubensis*, *P. lefebvrei,* and *P. pubescens* (see notes above for the first two). *Picoa pubescens* has dark reddish brown ascomata when young, becoming black when mature, with abundant persistent mycelial hairs; surface with irregular, low compound warts.

9. ***Picoa pubescens*** A. Paz, V. Kaounas, C. Agnello & C. Lavoise, *sp. nov*. MB 859757. Figure 3, Figure 4 and Figure 5 and Figure 11.
  *Etymology*: The Latin epithet “*pubescens*” means “covered with hairs”, in relation to the very abundant permanent mycelial hairs found on the surface of mature ascomata.
  *Type*: **Spain**, Euskadi, Álava, Labraza, soil, near *Pinus halepensis, Juniperus* sp., *Cistus albidus*, *Thymus vulgaris,* and *Calluna vulgaris*, 21 June 2013, *A. Paz* & *C. Lavoise* (BCN-myc 3591 holotype, IC21061307 isotype).
  Ascomata form globose stereothecia, 15–27 mm diam., dark reddish brown when young, becoming black when mature, with abundant and persistent mycelial hairs that are short and sparsely forked. Peridium (surface) with irregular compound warts measuring 126.5–200.5 µm (height) × 236.5–500.5 µm (base), with a crater-shaped center and 2–3 subtle vertical edges. Medullary excipulum (peridium section) with three distinct layers: (1) outer layer 53.5–91.5 µm thick, formed by 5–7 rows of cells at the apices and 3–4 rows at the bases, textura angularis, organized as a pseudoparenchymatous structure made of parallelepipedic cells, vertically parallel, 12–26.5 × 8.5–24.5 µm, Q = 1.31, walls up to 10 µm thick, reddish brown pigmented; this layer also has abundant bases of mycelial hairs, cylindrical, sometimes slightly wider at the base, measuring on average 22 × 18.5 µm; (2) intermediate layer 57–131.5 µm thick, textura angularis, with a pseudoparenchymatous structure made of parallelepipedic hyaline cells, vertically parallel, 15.5–32.5 × 10.5–19.5 µm, Q = 0.88; in this layer, there are also longer bifurcate and cylindrical cells that form the base of mycelial hairs; (3) internal layer (transition toward the gleba) 105.5–120.5 µm thick, textura intricate, with an intricate plectenchymatous structure made of increasingly smaller, elongated cells, 6.5–10.5 × 4–8 µm (long), Q = 0.64. Gleba composed of sterile zones with an intricate texture, made of elongated, bifurcate hyphae, with septa measuring 12 × 4.5 µm on average, and disordered fertile cells. Asci 125.5–*126*–128 × 62.5–*63.5*–64.5 µm, Q = 1.99, quickly evanescent, inamyloid, prototunicate, subglobose, with a peduncle 35–45 µm long (included in the ascus measurements), with thin walls, containing 6–8 ascospores. Ascospores broadly ellipsoidal, 24.5–*25*–25.5 × 22–*22.5*–23 µm, Q = 1.1–1.12, ornamented with minute granules, which join together forming very irregular semi-reticles.
  *Ecology and Distribution*: Collected in Spain, France, Greece, and Italy, always with the presence of *Cistus*, *Quercus,* or *Pinus*, in winter and spring.
  *Additional material studied*: **France**, Bouches-du-Rhône, under *Quercus ilex*, 13 March 1983, *G. Riousset* (ALV7017*). **Greece**, Artemis Attica, under *Cistus monspeliensis*, in forest with *Pinus halepensis*, *Pistacia lentiscus,* and *Quercus coccifera*, 22 February 19, *V. Kaounas* VK5561*; Rafina Attica, under *Pistacia lentiscus* in forest with *Pinus halepensis* and *Fumana* cf. *thymifolia*, 26 February 2019, *V. Kaounas* VK5565*. **Italy**, Puglia, Santo Antonio, under *Cistus* sp., 23 February 2013, *C. Agnello* CA23/02/2013*; Puglia, S. Antonio wood, S. Pancrazio, *Cistus* sp., 23 January 2013, *C. Agnello* CA23/01/2013*.
  *Notes*: *Picoa pubescens* is characterized by (1) dark reddish brown ascomata when young, becoming black when mature, with abundant and persistent mycelial hairs; (2) surface with irregular low compound warts (with 2–3 edges); (3) outer layer of the peridium is thick (3–7 rows of cells), pseudoparenchymatous, with parallelepipedic cells vertically parallel, with thick walls, up to 10 µm thick; (4) internal peridium layer is plectenchymatous, formed by elongated hyphae horizontally parallel; (5) ascospores broadly ellipsoidal, ornamented with minute granules that join together forming very irregular semi-reticles; (6) preference for neutral soils; (7) probably associated with *Cistus* and/or *Pinus*. The most closely related species are *P. cordubensis*, *P. lefebvrei,* and *P. llobregatina* (all of them are compared with *P. pubescens*, above).

10. ***Picoa* sect. *Communes*** P. Alvarado, A. Paz & Van Vooren, *sect. nov*. MB 859773. Figure 3, Figure 4 and Figure 5 and Figure 12, Figure 13, Figure 14, Figure 15, Figure 16, Figure 17, Figure 18, Figure 19, Figure 20, Figure 21 and Figure 22.
  *Etymology*: From the epithet of the type, *Picoa communis.*
  *Type*: *Picoa communis* A. Paz, C. Lavoise, L.-A. Parra, B. Vázquez & L. Barrio *sp. nov*.
  The section *Communes* includes species with (1) café-au-lait, brown, or black color; (2) surface with papillae, granules, or warts; (3) cells of the outer layer sclerenchymatous or parallelepipedic; (4) spherical to ellipsoidal ascospores, measuring up to 27 µm long, ornamented with isolated coarse or fine granules and sometimes with roughness; and (5) mainly associated with annual species of *Helianthemum* and maybe also *Tuberaria* or *Cistus*, sometimes with presence of *Pinus* or *Quercus.* The section is composed of *P. communis*, *P. fajardoi*, *P. montecchii*, *P. morarae*, *P. pacionii*, *P. papillata, P. pontica*, *P. truncata*, *P. sphaerospora,* and *P. vazqueziae*.

**Key to the sections of *Picoa* sect. *Communes***

1.Ascomata milky brown, surface with slightly darker tiny papillae.
*P. papillata.*
1.Ascomata brown or black, surface with larger granules or warts.22.Surface of the ascomata with rounded granules.32.Surface of the ascomata with angular warts.43.Surface of the ascomata with granules united by their bases, internal layer of the peridium intricate, plectenchymatous.*P. pacionii*.3.Surface of the ascomata with isolated granules, internal layer of the peridium pseudoparenchymatous, made of puzzle-like cells.*P. morarae*.4.Surface of the ascomata with simple angular warts.54.Surface of the ascomata with compound angular warts.65.Surface of the ascomata with warts very irregular in shape and size, mycelial hairs absent at maturity, inner layer of the peridium pseudoparenchymatous, with puzzle-shaped to slightly elongated cells.
*P. communis.*
5.Surface of the ascomata with pyramidal warts in the shape of a double-inverted triangle; mycelial hairs present at maturity, inner layer of the peridium plectenchymatous, with cells horizontally parallel to the outer layer.
*P. montecchii.*
6.Surface of the ascomata with black compound warts with a reddish-brown base and a truncated apex, outer and intermediate layers of the peridium without parallelepiped cells.
*P. truncata.*
6.Surface of the ascomata with compound warts without truncated apex.77.Ascospores spherical.
*P. sphaerica.*
7.Ascospores ellipsoidal.88.Ascomata very compact, black, resembling *Tuber aestivum.*
*P. vazqueziae.*
8.Ascomata not compact, reddish brown to dark brown.99.Ascomata reddish brown, outer and intermediate layer of the peridium with parallelepiped cells and internal layer plectenchymatous, not intricate.
*P. fajardoi.*
9.Ascomata dark brown, outer and intermediate layers of the peridium without parallelepiped cells and internal layer plectenchymatous, intricate.1010.Ascomata with mycelial hairs present at maturity, ascospores with a very thick perisporium and golden tones under the optical microscope.
*P. pontica.*
10.Ascomata with a few mycelial hairs when young, ascospores with a thin perisporium, hyaline.
*P. rodensis.*

**Figure 23:**
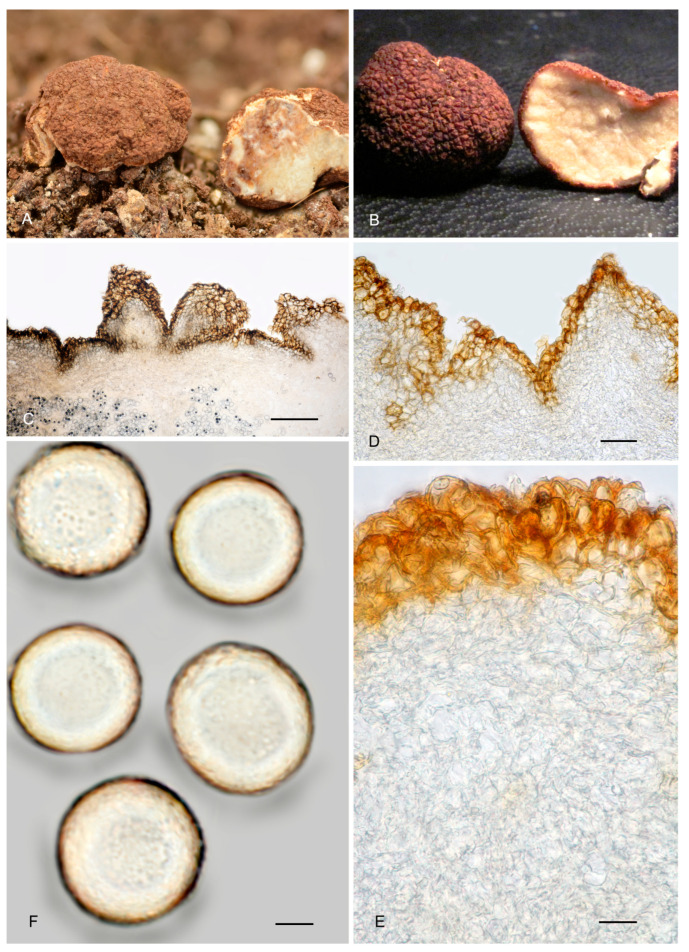
*Picoa microspora:* (**A**) ascomata BCN.myc 3614 (holotype); (**B**) ascomata GK3737; (**C**–**F**) BCN-myc 3614 (holotype): (**C**) section of peridium and gleba; (**D**,**E**) medullary excipulum with pseudoparenchymatous structure, made of subglobose or slightly elongated sclerenchymatous cells; (**F**) ascospores. Scale bars: (**C**) = 2 mm; (**D**) = 40 µm; (**E**) = 20 µm; (**F**) = 5 µm. Photos: (**A**) = A. Paz; (**B**) = G. Konstantinidis; (**C**–**F**) = A. Paz. Images (**A**,**B**) taken from dry material; (**C**–**F**) taken from rehydrated mounts.

11. ***Picoa communis*** A. Paz, C. Lavoise, L.-A. Parra, B. Vázquez & L. Barrio, *sp. nov*. MB 859758. Figure 3, Figure 4 and Figure 5 and Figure 12.
  *Etymology*: The Latin epithet “*communis*” means common and refers to its abundance among collections found in Spain.
  *Type*: **Spain**, Castilla y León, Palencia, meadow with *Tuberaria guttata* and *Helianthemum* sp., 25 April 2016, *L.-A. Parra*, *C. Lavoise*, *C. Rojo* & *A. Paz* (BCN-myc 3592 holotype, IC25041601 isotype).
  Ascomata form subglobose stereothecia, up to 45 mm diam., with surface sometimes cracked, reddish brown to dark brown with age, initially covered with abundant reddish brown mycelial hairs, disappearing with age or handling. Peridium (surface) forms simple warts, very irregular in shape and size, 185–350 (height) × 310–540 µm (base). Medullary excipulum (peridium section) arranged in three layers: (1) outer layer 32.5–116 µm thick, textura angularis, with a pseudoparenchymatous structure, made of globose to polyhedrical sclerenchymatous cells measuring 30 × 27.5 µm on average, Q = 1.12, walls up to 4.2 µm thick, intensely brown pigmented; peridium cells form 1–2 rows at the base of warts and 3–4 at the apices; young ascomata have hairs in the outer peridium layer, 230–350 × 8–11 µm, with rounded tips and inflated bases about 39–45 µm wide, septa measuring 35–55 µm long; (2) intermediate layer 42.5–74 µm thick, textura angularis, with a pseudoparenchymatous structure, made of subglobose to polyhedrical sclerenchymatous cells, hyaline, and measuring on average 29 × 24 µm, Q = 1.19, with increasingly thinner walls, up to 2.1 µm thick; (3) inner layer 169.5–311 µm thick, textura angularis, with a pseudoparenchymatous structure, made of cells that become irregular and puzzle-shaped in deep; these cells are interspersed with glebal tissue formed by interwoven hyphae. Gleba composed of sterile zones with an intricate texture, made of elongated, bifurcate hyphae with septa measuring 11 × 3.5 µm on average, and disordered fertile areas with asci. Asci 153–*154.5*–156.5 × 73.5–*74.5*–77 µm, quickly evanescent, inamyloid, prototunicate, subglobose, with a peduncle 45–65 µm long (included in the ascus measurements), thin-walled and simple, containing 6–8 ascospores. Ascospores globose to slightly ovoid, 23.5–*25.5*–27.5 × 21.5–*23*–24.5 µm, Q = 0.99–1.12, ornamented with abundant large warts measuring on average 0.8 µm (base) × 1.2 µm (height).
  *Ecology and Distribution*: So far found in France, Spain, and Turkey, frequently in ancient threshing floors with sandy and stony soils, near *Helianthemum* sp. Also found in fixed secondary dunes with *Helianthemum* spp. *Pinus halepensis*, *P. pinaster*, *Quercus coccifera*, *Asphodelus albus*, *Cistus monspeliensis*, *C. salvifolius*, *Tuberaria guttata*, and *Juniperus* spp. from January to June.
  *Additional material studied*: **France**, Tarn, February 2006, *T. Sánchez* (AH 39205*). **Spain**, Cantabria, Liébana, Caloca, meadow with *Helianthemum* sp., 7 May 2005, *A. Paz* BCN-myc 3593, IC07050508; ibid. 23 April 2007, *A. Paz* IC23040715. Castilla y León, Ávila, Narrillos de San Leonardo, meadow with *Helianthemum* sp., 5 May 2021, *L. A. Trujillo* LTP2021050501; Burgos, Abajas, under *Quercus ilex*, 6 April 2007, *F. Sáinz* PSS086.001A*; Palencia, Olmos de Ojeda, meadow with *Helianthemum* sp., 3 April 2017, *L. Barrio* BCN-myc 3594, IC03041715; Valladolid, Aldeamayor de San Martín, near *Pinus pinaster* and *Helianthemum* sp., 14 May 1994, *A. García-Blanco*, *M. Sanz* & *J. B. del Val* AVM998*; Valladolid, Traspinedo, vineyard, 19 May 1996, *A. García Blanco*, *M. Sanz* & *J.B. del Val* AVM1490; Valladolid, Urueña, ancient threshing floor with abundant *Helianthemum* sp., 26 April 2006, *A. García Blanco*, *M. Sanz* & *J.B. del Val* AVM2446*; ibid. calcareous plain with *Helianthemum* sp., 17 April 2008, *J. Cabero* JC080417BT; ibid. 3 May 2021, *B. Vázquez* IC03052102;. **Turkey**, Baskil, Kadiköy–Elazigh, on sandy and stony soils, 8 May 1985, *A.F. Güan* n. 511 (AQUI9324).
  *Notes*: *Picoa communis* is characterized by (1) ascomata reddish brown to dark brown with age, mycelial hairs absent at maturity; (2) surface with irregular, slightly prominent warts; (3) outer layer of the peridium with cells up to 30 µm wide; (4) inner layer of the peridium pseudoparenchymatous, with puzzle-shaped to slightly elongated cells; (5) ascospores globose to slightly ovoid, ornamented with abundant large granules; (6) preference for sandy or stony neutral soils; (7) probably associated with annual species of *Helianthemum*, maybe also *Pinus*, *Quercus*, *Cistus* and/or *Tuberaria*. The most closely related species are *P. fajardoi*, *P. montecchii*, *P. morarae*, *P. pacionii*, *P. papillata*, *P. pontica*, *P. rodensis*, *P. sphaerospora*, *P. truncata*, and *P. vazqueziae. Picoa fajardoi* is distinguished by its outer and intermediate layers of the peridium formed by parallelepipedic cells; inner layer of the peridium plectenchymatous; and preference for calcareous soils. *Picoa montecchii* has a surface covered with simple pyramidal warts shaped like a double-inverted triangle; permanent mycelial hairs at maturity; inner layer of the peridium plectenchymatous; and preference for calcareous soils. *Picoa morarae* has a surface with pronounced granules lacking halo; internal peridium made of polyhedrical cells interspersed with elongated hyphae; and ascospores slightly more ellipsoidal (Q = 1.15). *Picoa pacionii* has a surface with very fragile, irregular granules united by their bases; a slightly thinner intermediate peridium layer (31.5–41.5 µm thick), made of elongated cells; and ascospores slightly more ellipsoidal (Q = 1.19). *Picoa papillata* has milky brown ascomata with slightly darker papillae; ascospores slightly more ellipsoidal (Q = 1.15), with a rough ornamentation; and preference for calcareous soils. *Picoa pontica* is distinguished by its surface with isolated and not very pronounced compound pyramidal warts and mycelial hairs present at maturity; internal layer of the peridium plectenchymatous, intricate, made of elongated hyphae; ascospores ornamented with small ridges and isolated tiny granules; and preference for calcareous soils. *Picoa rodensis* has a surface with very pronounced, isolated, compound pyramidal warts; outer layer of the peridium made of cells with walls up to 13.7 µm thick; internal peridium layer plectenchymatous, intricate; ascospores ornamented with abundant, small but pronounced granules and isolated small ridges; and preference for calcareous soils. *Picoa sphaerospora* has an inner layer of the peridium made of puzzle-shaped cells interspersed with elongated hyphae arranged in parallel and spherical ascospores 23–24 µm in diam. *Picoa truncata* has a surface covered with compound truncated pyramidal warts, with a reddish base; intermediate layer of the peridium pseudoparenchymatous, made of very small globose to polyhedric cells, 11.5 × 12 µm on average; and preference for calcareous soils. Finally, *P. vazqueziae* has black-colored ascomata, covered with very large compound pyramidal warts; intermediate and internal layers plectenchymatous, with an intricate texture; and preference for calcareous soils.

12. ***Picoa fajardoi*** A. Paz, E.-J. Salvador & C. Lavoise, *sp. nov*. MB 859759. Figure 3, Figure 4 and Figure 5 and Figure 13.
  *Etymology*: Dedicated to José Fajardo Rodríguez, professor at the Popular University of Albacete, for his dedication and commitment to the rural world and nature.
  *Type*: **Spain**, Castilla y León, Burgos, Mecerrelles, mixed *Pinus* and *Quercus faginea* forest with *Cistaceae*, 2 April 2015, *A. Paz* & *C. Lavoise* (BCN-myc 3595 holotype, IC20041515 isotype).
  Ascomata form lobed stereothecia, up to 30 mm diam., reddish brown (intense when young, darker when mature). Peridium (surface) covered by irregular compound warts frequently showing 2–4 edges and a semi-truncated apex, measuring 82–394.5 µm (height) × 238–586.5 µm (base), Q = 0.51, with abundant but fragile mycelial hairs that disappear at maturity. Medullary excipulum (peridium section) arranged in 3 layers: (1) outer layer measuring 37 µm (at the bases of warts)–84 µm (at the apices of warts) thick, textura angularis, with a pseudoparenchymatous structure, made of elongated to parallelepipedic cells, measuring on average 19.5 × 12.5 µm, Q = 1.58, walls up to 3.3 µm thick, brown-pigmented; these cells form 1–2 rows of at the base of warts and 2–3 rows at the apices; (2) intermediate layer measuring 75 µm (at the bases of warts) to 181.5 µm (at the apices) thick, textura angularis, pseudoparenchymatous, made of parallelepipedic to very irregular hyaline cells, measuring 12.5 × 16.5 µm on average, Q = 0.77; (3) internal layer (transition toward the gleba) measuring 167.5 µm (at the bases of warts) to 231.5 µm (at the apices) thick, plectenchymatous, made of elongated cells, horizontally parallel, measuring on average 14 × 8.5 µm, Q = 1.69. Gleba with sterile zones in an intricate texture, made of bifurcate, elongated hyphae with septa measuring 10 × 3.5 µm on average, as well as disordered fertile areas containing asci. Asci 165–*167.5*–170 × 76.5–*77.5*–80 µm, quickly evanescent, inamyloid, prototunicate, subglobose, with a thin simple wall, peduncle 45–60 µm long (included in the ascus measurements), containing 4–8 ascospores. Ascospores broadly ellipsoidal, 24.5–*26.5*–27.5 × 21.5–*23*–25 µm, Q = 1.12–1.15, ornamented with abundant, large and isolated granules.
  *Ecology and Distribution*: Collected only in central Spain (Castilla y León: Burgos and Valladolid; Castilla-La Mancha: Cuenca), in sandy, calcareous soils, near *Helianthemum* and other *Cistaceae*, in forest clearings.
  *Additional material studied*: **Spain**, Castilla-La Mancha, Cuenca, Casa de los Pinos, calcareous soil with *Helianthemum salicifolium*, *Tuberaria guttata*, *Rosmarinus officinalis* and isolated *Quercus* sp., 3 March 2017, *E–J Salvador* BCN-myc 3596*, IC30031721; Castilla y León, Burgos; Solduengo, sandy soil with *Cistus salvifolius*, *Tuberaria guttata* and *Helianthemum apenninum*, 3 May 2008, *P. Sainz* PSS-068.05*; ibid. 4 May 2013, PSS-068.10*; Valladolid, Castromonte, La Espina, sunny area, calcareous soil, near *Helianthemum* sp., 23 April 2003, *A. Paz* & *C. Lavoise* BCN-myc 3597*, IC23040321; ibid. 25 April 2007, *A. García* AVM2772*.
  *Notes*: *Picoa fajardoi* is characterized by (1) mycelial hairs absent at maturity; (2) surface showing irregular low compound warts with 2–4 edges; (3) outer layer of the peridium very thin, pseudoparenchymatous, made of 1–3 rows of parallelepipedic cells, with walls up to 3.3 µm thick; (4) inner layer of the peridium very thick, plectenchymatous, made of horizontal parallel elongate cells; (5) ascospores broadly ellipsoidal, ornamented with abundant isolated large warts; (6) preference for calcareous soils; (7) probably associated with annual species of *Helianthemum*, maybe also other Cistaceae plants. The more closely related species are *P. communis* (see above), *P. montecchii*, *P. morarae*, *P. pacionii*, *P. papillata*, *P. pontica*, *P. rodensis*, *P. sphaerospora*, *P. truncata,* and *P. vazqueziae. Picoa montecchii* has a surface covered with simple pyramidal warts with the shape of a double-inverted triangle and mycelial hairs present at maturity and intermediate and inner layers lacking parallelepipedic cells. *Picoa morarae* has tawny brown to intensely reddish ascomata with dark, pronounced granules, internal peridium layer pseudoparenchymatous, preference for neutral soils, and slightly more ellipsoid ascospores (Q = 1.15), ornamented with small ridges that join together forming alveoles or semi-reticles. *Picoa pacionii* has a surface with very fragile granules united by their bases; outer and intermediate layers without parallelepipedic cells; preference for neutral soils. *Picoa papillata* has milky brown ascomata covered with slightly darker papillae; outer and intermediate layers without parallelepipedic cells; and slightly more ellipsoid ascospores (Q = 1.15), with a rough ornamentation. *Picoa pontica* has a surface covered with compound pyramidal warts and abundant and permanent mycelial hairs, ascospores ornamented with small ridges and tiny isolated granules, with a perisporium showing golden tones under the optical microscope. *Picoa rodensis* has dark brown to almost black ascomata, covered with pronounced compound pyramidal warts, outer and intermediate layers without parallelepipedic cells, and ascospores ornamented with abundant small, pronounced granules and isolated small ridges. *Picoa sphaerospora* has intermediate and inner layers of the peridium pseudoparenchymatous, made of puzzle-shaped cells; spherical ascospores; smaller, 23–24 µm diam.; and preference for neutral soils. *Picoa truncata* has prominent compound pyramidal black warts, with a truncated apex and a reddish-brown base; the outer and intermediate layers of the peridium lack parallelepipedic cells. Finally, *Picoa vazqueziae* produces black, very compact ascomata resembling *Tuber aestivum*, with very large compound pyramidal warts; intermediate peridium layer plectenchymatous, intricate, mixed with small globose to polyhedric cells.

13. ***Picoa montecchii*** A. Paz, C. Lavoise, E.J. Salvador-Fernández, J.L. Escobar & J.M. Vidal, *sp. nov*. MB 859760. Figure 3, Figure 4 and Figure 5 and Figure 14.
  *Etymology*: Dedicated to the great Italian mycologist Amer Montecchi, a dear friend of the authors, and a worldwide reference in the study of hypogeous fungi.
  *Typus*: **Spain**, Castilla-La Mancha, Albacete, Vandelaras de Abajo, next to *Helianthemum salicifolium*, calcareous soil, 27 February 2021, *E. J. Salvador-Fernández* & *J. L. Escobar-Atienza* (BCN-myc 3598 holotype, IC27022103 isotype).
  Ascomata form globose or slightly lobed stereothecia, up to 30 mm diameter, dark reddish brown to dark brown or almost black with age, covered with abundant and quite persistent mycelial hairs, septate, encrusted, reddish brown. Peridium (surface) with prominent pyramidal warts, often shaped like a double-inverted triangle, 567.5 µm (height) × 280 µm (base). Medullary excipulum (peridium section) with 3 distinct layers: (1) outer layer 24.5–55.5 thick, textura angularis, pseudoparenchymatous, made of subglobose sclerenchymatous cells, measuring 24 × 21 µm on average, Q = 1.14, walls up to 4.5 µm thick, dark brown; peridium cells form 1–2 rows at the base of warts and 2–3 rows at the apices; this layer also has the bases of the external mycelial hairs, made of elongated, cylindrical cells, measuring on average 29.5 × 18 µm; (2) intermediate layer 107.5–253 µm thick, textura angularis, pseudoparenchymatous, made of progressively more irregular and elongated hyaline cells, measuring on average 18.5 × 10.5 µm, Q = 1.95, thin-walled; these cells are interspersed also with elongated and cylindrical cells (sometimes bifurcate) at the base of external mycelial hairs; (3) inner layer measuring 138 µm (at the bases of warts) to 249.5 µm (at the apices) thick, plectenchymatous, made of variably sized, elongated, and septate cells, horizontally parallel; these cells are interspersed with interwoven and forked hyphae of the gleba. Gleba showing sterile zones with an intricate texture, made of elongated, bifurcate hyphae, with septa of 9.5 × 4 µm on average, and disordered fertile areas with asci. Asci 172–*173*–174.5 × 75.5–*76*–77.5 µm, quickly evanescent, inamyloid, prototunicate, subglobose, peduncle 50–65 µm long (included in the ascus measurements), thin-walled and simple, containing 6–8 ascospores. Ascospores globose to slightly ovoid, 22.5–*25.5*–27.5 × 20.5–*23*–24.5 µm, Q = 0.97–1.12, ornamented with very abundant large granules.
  *Ecology and Distribution*: Collected only in Spain, in sandy, calcareous soils, near annual species of *Helianthemum* (i.e., *H. salicifolium*, *H. apenninum*), sometimes with the presence of *Cistus* spp. (i.e., *C. salvifolius*, *C. albidus*, *C. monspeliensis*), *Tuberaria guttata,* and other plants.
  *Additional material studied*: **Spain**, Castilla y León, Burgos, Llanos de Bureba, sandy soil with *Cistus salvifolius*, *Tuberaria guttata* and *Helianthemum apenninum*, 9 January 2012, *P. Sainz* PSS–068.09*; Valladolid, Tiedra, calcareous plain, near *Quercus ilex*, 3 April 2011, *J. Cabero* JC110403BT; Catalonia, Girona, Mas Julià, sandy soil with *Helianthemum* sp., *Cistus albidus*, *C. monspeliensis* and *Asphodelus albus*, near *Pinus halepensis* and *Quercus coccifera*, 15 March 1991, *J. M. Vidal* JMV 910315-3, BCN-myc 3599*. Torroella de Montgrí, Platja de la Fonollera, dunes near the beach, with *Helianthemum apenninum* subsp. *pilosum*, 11 March 2001, *JM Vidal* JMV20010311-1*.
  *Notes*: *Picoa montecchii* is characterized by having (1) mycelial hairs present at maturity; (2) surface covered with simple pyramidal warts having a shape of a double-inverted triangle; (3) outer layer of the peridium very thin (1–3 rows of cells), pseudoparenchymatous, made of sclerenchymatous subglobose and elongated cells, not exceeding 25 µm long; (4) inner layer of the peridium plectenchymatous, made of elongated, bifurcate cells, horizontally parallel; (5) ascospores subglobose to ovoid, ornamented with abundant large granules; (6) preference for calcareous soils; (7) probably associated with annual species of *Helianthemum* spp. and maybe also other Cistaceae. The most closely related species are *P. communis*, *P. fajardoi*, *P. morarae*, *P. pacionii*, *P. papillata*, *P. pontica*, *P. rodensis*, *P. sphaerospora*, *P. truncata,* and *P. vazqueziae.* The first two species are compared with *P. montecchii* above. Ascomata of *Picoa morarae* have a surface covered with pronounced granules and mycelial hairs only present in youth; internal layer is pseudoparenchymatous, ascospores slightly more ellipsoidal (Q = 1.15), ornamented with small ridges that join together forming alveoles or semi-reticles, and preference for neutral soils. *Picoa pacionii* has a surface covered by very fragile granules united by their bases, without mycelial hairs; ascospores ellipsoidal, Q = 1.19, and preference for neutral soils. *Picoa papillata* has milky brown ascomata, covered with slightly darker papillae; ascospores slightly more ellipsoidal (Q = 1.15), and preference for gypsum soils. *Picoa pontica* has a surface with compound pyramidal warts; ascospores ornamented with small ridges and tiny isolated granules, with a perisporium with golden tones under the light microscope. *Picoa rodensis* has a surface with compound pyramidal warts, covered with a few mycelial hairs in youth, and ascospores ornamented with abundant small, pronounced granules and isolated small ridges. *Picoa sphaerospora* is covered with prominent, irregular compound warts, with mycelial hairs only present in youth; inner layer of the peridium is pseudoparenchymatous; ascospores are spherical and a bit smaller, 23–24 µm diam., and has preference for neutral soils. *Picoa truncata* is covered with black warts with a truncated apex and a reddish-brown base, with mycelial hairs only present in youth. Finally, *P. vazqueziae* has black, very compact ascomata with very large compound pyramidal warts and a plectenchymatous intricate intermediate layer.

14. ***Picoa morarae*** A. Paz, A. Zambonelli & C. Lavoise, *sp. nov*. MB 859762. Figure 3, Figure 4 and Figure 5 and Figure 15.
  *Etymology*: Dedicated to the great Italian mycologist Marco Morara, a dear friend of the authors and tireless scholar specialized in hypogeous fungi.
  *Type*: **Turkey**, Cappadocia, under *Cistus* sp., May 2011, *A. Zambonelli* & *I. Ozdemir* Z. 4246, (BCN-myc 3600 holotype, IC01051134 isotype).
  Ascomata form subglobose or lobed stereothecia, up to 30 mm diam., tawny brown to intensely reddish, showing mycelial hairs in youth. Peridium (surface) with dark, pronounced granules measuring 394–494.5 µm (height) × 254.5–456 µm (base), Q = 1.34, without halo. Medullary excipulum (peridium section) arranged in three layers: (1) outer layer textura angularis, pseudoparenchymatous, 47–59.5 µm thick, formed by 2–3 rows of cells at the apices and 1–2 rows at the bases, with globose, sclerenchymatous cells, 12.5–30 × 12.5–27.5 µm, Q = 1.07, walls up to 8.2 µm thick, dark brown pigmented; this layer has also scattered cylindrical bases of mycelial hairs, 22–35.5 × 15–22.5 µm, Q = 1.56; (2) intermediate layer 46–151.5 µm thick, textura angularis, pseudoparenchymatous, made of hyaline, globose to polyhedrical cells, measuring 17.5–29.5 × 17–27 µm, Q = 1.02, walls up to 3 µm; (3) internal layer (transition toward the gleba) textura angularis, pseudoparenchymatous, measuring 215–414 µm thick, made of increasingly elongated angular to puzzle-like cells, 10–20 × 6.5–15.5 µm, Q = 1.55. Gleba composed of sterile zones with an intricate texture, formed by elongated, bifurcate hyphae, with septa measuring 11 × 4.5 µm on average, as well as disordered fertile areas with asci. Asci 159.5–*161*–163 × 70–*70.5*–72 µm, quickly evanescent, inamyloid, prototunicate, subglobose, peduncle 45–55 µm long (included in the ascus measurements), thin-walled and simple, containing 6–8 ascospores. Ascospores ellipsoidal, 23.5–*25*–26.5 × 20.5–*21.5*–23 µm, Q = 1.1–1.15, ornamented with small ridges that join together forming alveoles or semi-reticles.
  *Ecology and Distribution*: Collected so far in Algeria and Turkey, under *Cistus* and annual *Helianthemum*, in sandy neutral soil, in spring.
  *Additional material studied*: **Algeria**, Tiaret, Benhamad, in sandy, basic soil, near *Helianthemum hirtum*, February 2009, BMBH1*; Ibid. BMBH3*.
  *Notes*: *Picoa morarae* is characterized by (1) ascomata tawny brown to intensely reddish, with mycelial hairs absent at maturity; (2) surface with pronounced granules lacking halo; (3) outer layer of the peridium very thin (1–2 rows of cells), pseudoparenchymatous, made of globose sclerenchymatous cells, with walls up to 8.2 thick; (4) internal peridium layer very thick, pseudoparenchymatous, made of polyhedrical cells interspersed with elongated hyphae; (5) ellipsoidal ascospores, ornamented with small ridges that join together forming alveoles or semi-reticles; (6) preference for neutral soils; (7) probably associated with annual species of *Helianthemum* or *Cistus* spp. The most closely related species are *P. communis*, *P. fajardoi*, *P. montecchii*, *P. pacionii*, *P. papillata*, *P. pontica*, *P. rodensis*, *P. sphaerospora*, *P. truncata,* and *P. vazqueziae.* The first three are compared with *P. morarae* above. *Picoa pacionii* has an intermediate peridium layer made of elongated, hyaline sclerenchymatous cells; internal layer arranged as an intricate plectenchymatous structure and more ellipsoid ascospores (Q = 1.19), ornamented with small but prominent isolated granules. *Picoa papillata* has milky brown ascomata, with slightly darker papillae; outer peridium layer made of very large subglobose to elongated cells, 31.5–45 × 30.5–71 µm; preference for calcareous gypsum soils, and ascospores with rough ornamentation not forming alveoli or semi-reticles. *Picoa pontica* has reddish brown ascomata when young, becoming almost black when fully mature, covered with compound pyramidal warts, with abundant and permanent mycelial hairs; preference for calcareous soils, and ascospores perisporium showing golden tones under the optical microscope. *Picoa rodensis* has dark brown to almost black ascomata covered with pronounced compound pyramidal warts; internal layer of the peridium plectenchymatous, intricate; preference for calcareous soils. *Picoa sphaerospora* has the intermediate and inner layers of the peridium made of puzzle-shaped cells, and ascospores spherical, 23–24 µm diam. The surface of *Picoa truncata* is covered with black, prominent, compound pyramidal warts, with truncate apices and reddish-brown bases; internal layer peridium plectenchymatous, intricate; ascospores ornamented with large, isolated granules, and preference for calcareous soils. *Picoa vazqueziae* has black, very compact ascomata, covered with very large compound pyramidal warts; intermediate layer plectenchymatous, intricate; ascospores with rough ornamentation, not forming alveoli or semi-reticles, and preference for calcareous soils.

15. ***Picoa pacionii*** A. Paz & C. Lavoise, *sp. nov*. MB 859763. Figure 3, Figure 4 and Figure 5 and Figure 16.
  *Etymology*: Dedicated to the great Italian professor Giovanni Pacioni, a dear friend of the authors and respected expert in hypogeous fungi.
  *Typus*: **Turkey**, Baskil, Harabakayis, in sandy and stony soils, 22 April 1984, *F. Güan* n. 517 (BCN-myc 3601 holotype, AQUI9325 isotype).
  Ascomata form subglobose stereothecia, up to 30 mm diam., light reddish brown to reddish brown in old age, without mycelial hairs. Peridium (surface) with very fragile granules that break if brushed, united by their bases, irregular in size and shape, measuring 115.5–391.5 µm (height) × 348–896 µm (base). Medullary excipulum (peridium section) arranged in three layers: (1) outer layer textura angularis, pseudoparenchymatous, made of very small subglobose to elongated sclerenchymatous cells, 16–28 × 14.5–34.5 µm, Q = 1.02, with walls up to 7.7 µm thick, reddish brown pigmented, arranged in 1–2 rows at the bases and at the apices of the granules; (2) intermediate layer 31.5–41.5 µm thick, textura angularis, pseudoparenchymatous, made of elongated, hyaline, sclerenchymatous cells, 7.5–14 × 11.5–24.5 µm, Q = 0.64, with increasingly thinner walls up to 2.9 µm thick; (3) internal layer (transition toward the gleba) textura intricate, 147.5–411.5 µm thick, showing an intricate plectenchymatous structure, made of very irregular hyphae, 6.5–15.5 × 4.5–11.5 µm, Q = 1.41, decreasing in size near the gleba, where it is found interspersed with glebal tissue of interwoven hyphae. Gleba composed of sterile zones with an intricate texture, made of elongated, bifurcate hyphae, with septa of 12 × 4.5 µm on average, as well as disordered fertile areas containing asci. Asci 135.5–*136*–139 × 64.5–*65*–67 µm, quickly evanescent, inamyloid, prototunicate, subglobose, peduncle 55–75 µm long (included in the ascus measurements), with a thin and simple wall, containing (3–) 4–8 ascospores. Ascospores ellipsoidal, 24.5–*26.5*–28 × 20.5–*22*–23.5 µm, Q = 1.16–1.20, ornamented with small but prominent isolated granules.
  *Ecology and Distribution*: Collected in Algeria, Iran [32], and Turkey, in nutrient-poor, sandy, and stony soils, probably associated with annual species of *Helianthemum*, fruiting in spring.
  *Additional material studied*: **Algeria**, Tiaret, Benhamed, near *Helianthemum hirtum*, February 2009 (BMBH2*). Iran, Fars, 27 March 2011, *S. Jamali* (AH39286*).
  *Notes*: *Picoa pacionii* is characterized by (1) mycelial hairs absent at maturity; (2) surface with very fragile, irregular granules, united by their bases; (3) outer layer of the peridium very thin (1–3 rows), pseudoparenchymatous, made of subglobose and elongated sclerenchymatous cells, with walls up to 7.7 µm thick; (4) intermediate peridium layer very thin, pseudoparenchymatous, made of elongated cells, measuring 11 × 18 µm; (5) asci containing (3–) 4–8 ascospores, (6) ascospores subglobose to broadly ellipsoidal, ornamented with fine, pronounced, isolated granules; (6) preference for neutral soils; (7) probably associated with annual species of *Helianthemum* spp. The most closely related species are *P. communis*, *P. fajardoi*, *P. montecchii*, *P. morarae*, *P. papillata*, *P. pontica*, *P. rodensis*, *P. sphaerospora*, *P. truncata,* and *P. vazqueziae.* The first four are compared with *P. pacionii* above. *Picoa papillata* has milky brown ascomata with slightly darker papillae; ascospores slightly more ellipsoid (Q = 1.15), with a rough ornamentation, and preference for calcareous gypsum soils. *Picoa pontica* has reddish brown ascomata when young, becoming almost black when fully mature, covered with compound pyramidal warts, as well as abundant and permanent mycelial hairs; ascospores ornamented with small ridges and tiny isolated granules, with golden tones in the perisporium under the optical microscope, and preference for calcareous soils. *Picoa rodensis* has dark brown to almost black ascomata, covered with pronounced compound pyramidal warts; ascospores ornamented with abundant, small, pronounced granules and isolated small ridges, and preference for calcareous soils. *Picoa sphaerospora* has reddish brown to dark brown ascomata, or almost black at the end, covered with prominent, irregular compound warts; intermediate and inner layers of the peridium pseudoparenchymatous, made of puzzle-shaped cells, and spherical ascospores, 23–24 µm diam. *Picoa truncata* is covered with black prominent compound pyramidal warts showing a truncated apex and a reddish-brown base; ascospores slightly more globose (Q = 1.11), and preference for calcareous soils. Finally, *P. vazqueziae* has black and very compact ascomata, covered with very large compound pyramidal warts; intermediate layer plectenchymatous and intricate; and preference for calcareous soils.

16. ***Picoa papillata*** A. Paz, C. Lavoise, J. A. Martínez & J. M. Martínez, *sp. nov.* MB 859764. Figure 3, Figure 4 and Figure 5 and Figure 17.
  *Etymology*: From Latin *papillata*, derived from *papilla* meaning “small button”, referring to the small warts (or papillae) present on the surface of the ascomata.
  *Type*: **Spain**, Castilla La Mancha, Cuenca, Belinchón, in gypsum soils, with annual *Helianthemum* spp. plants, 4 April 2017, *J. A. Martínez* & *J. M. Martínez* (BCN-myc 3602 holotype; IC4041702 isotype).
  Ascomata form subglobose stereothecia, up to 23 mm diam., milky brown, with remnants of mycelial hairs. Peridium (surface) with slightly darker papillae, measuring 143.5–188 µm (height) × 452–535 µm (base). Medullary excipulum (peridium section) with three distinct layers: (1) outer layer 74–105.5 µm thick, sometimes almost absent (since the papillae are isolated and break easily), textura angularis, pseudoparenchymatous, made of very large subglobose to elongated cells (the largest of the genus), arranged in two rows at the apices of the papillae and one row at the bases, 31.5–45 × 30.5–71 µm, Q = 0.90, slightly light brown pigmented, and with a very thick wall, up to 8.3 µm thick; (2) intermediate layer 92–125.5 µm thick, textura angularis, pseudoparenchymatous, made of very irregular, subglobose to elongated hyaline cells, 20–53 × 22–54.5 µm, Q = 0.88, with increasingly thinner walls, up to 4.6 µm thick, interspersed by segmented hyphae; (3) internal layer (transition toward the gleba) 185.5–235 µm thick, plectenchymatous, made of the most elongated, segmented hyphae, arranged in parallel with respect to the gleba, 7.5–11.5 × 17.5–18.5 µm, Q = 1.62. Gleba composed of sterile zones with an intricate texture, made of elongated, bifurcate hyphae, with septa measuring 10.5 × 3.5 µm on average, as well as disordered fertile areas with asci. Asci 139.5–*142*–144 × 65.5–*67*–70 µm, quickly evanescent, inamyloid, prototunicate, subglobose, peduncle 55–70 µm long (included in the ascus measurements), with a thin simple wall, containing (3–) 4–8 ascospores. Ascospores ellipsoidal, 21.5–*25*–26.5 × 19.5–*21.5*–23 µm, Q = 1.12–1.16, with a rough ornamentation.
  *Ecology and Distribution*: So far found in Algeria and Spain, in gypsum, calcareous, and sandy soils, near annual species of *Helianthemum*, in spring.
  *Additional material studied*: **Algeria**, Tiaret, Benhamed, in sandy basic soil, near *Helianthemum hirtum*, February 2009, BMBH7+; ibid. BMBH8*; Tiaret, Bouchouat, March 2013, BMBC15*. **Spain**, Aragón, Zaragoza, Bujaraloz, 21 March 2004, *M. Obregón* (AH37801*).
  *Notes*: *Picoa papillata* is characterized by (1) the absence of mycelial hairs at maturity; (2) a café-au-lait surface with darker papillae; (3) an outer layer of the peridium very thin or absent (0–2 rows), pseudoparenchymatous, made of very large subglobose sclerenchymatous cells, with walls up to 8.3 µm thick; (4) an inner layer of the peridium very thick, plectenchymatous, made of elongated hyphae, horizontally parallel; (5) asci containing (3–) 4–8 broadly ellipsoidal ascospores with rough ornamentation; (6) preference for calcareous gypsum soils; (7) associated with annual species of *Helianthemum* spp. The most closely related species are *P. communis*, *P. fajardoi*, *P. montecchii*, *P. morarae*, *P. pacionii*, *P. pontica*, *P. rodensis*, *P. sphaerospora*, *P. truncata,* and *P. vazqueziae.* The first five are compared with *P. papillata* above. *Picoa pontica* has reddish brown ascomata when young to almost black when fully mature, covered with compound pyramidal warts, and abundant and permanent mycelial hairs, as well as ascospores ornamented with small ridges and tiny isolated granules, having a perisporium with golden tones under the optical microscope. *Picoa rodensis* has dark brown to almost black ascomata, covered with pronounced compound pyramidal warts, and ascospores ornamented with abundant small, pronounced granules and isolated small ridges. *Picoa sphaerospora* has reddish brown to dark brown ascomata that turn almost black at the end, covered with prominent, irregular compound warts; ascospores spherical, 23–24 µm diam., and preference for neutral soils. *Picoa truncata* is covered by black prominent compound pyramidal warts, with truncated apices and reddish-brown bases; ascospores slightly more globose (Q = 1.11). Finally, *P. vazqueziae* has black and very compact ascomata, covered with very large compound pyramidal warts, as well as a plectenchymatous, intricate intermediate peridium layer.

17. ***Picoa pontica*** M. Slavova, B. Assyov & A. Paz, *sp. nov.* MB 859765. Figure 3, Figure 4 and Figure 5 and Figure 18.
  *Etymology*: From the Latin name *Pontus Euxinus*, the ancient name of the Black Sea.
  *Type*: **Bulgaria**, Klementevo, Varna, under *Helianthemum nummularium*, *Carpinus* and *Quercus*, in calcareous soil, 22 May 2019, *M. Slavova* & *B. Assyov* (BCN-myc 3603 holotype, 2341F3475 isotype).
  Ascomata form globose or slightly lobed stereothecia, 20–35 mm diam., reddish brown when young to almost black when fully mature, with abundant and permanent mycelial hairs. Peridium (surface) with compound pyramidal warts, variable in size, measuring 236.5–374.5 µm (height) × 435–631 µm (base), with 4–8 well-pronounced vertical edges. Medullary excipulum (peridium section) arranged in three layers: (1) outer layer 23.5–64 µm thick, formed by 2–3 rows of cells at the apices and 1–2 at the bases, textura angularis, pseudoparenchymatous, made of subglobose or slightly elongated cells, 18.5–30 × 23.5–38.5 µm, Q = 0.79, with walls up to 9.1 µm thick, reddish brown pigmented, interspersed with the bases of the mycelial hairs that are cylindrical, slightly wider toward the bases, measuring on average 25.5 × 15.5 µm, Q = 1.73; (2) intermediate layer 127–217 µm thick, textura angularis, pseudoarenchymatous, made of hyaline, globose to polyhedrical cells, 15.5–32.5 × 15–32 µm, Q = 0.99; (3) internal layer (transition toward the gleba) textura intricate, 181.5–215.5 µm thick, plectenchymatous, made of increasingly smaller, elongated cells, intrincate, 11.5–26.5 × 6.5–13.5 µm, Q = 0.56. Gleba has sterile zones with an intricate texture, made of elongated, bifurcate hyphae, with septa measuring on average 11.5 × 4.5 µm, as well as disorganized fertile areas with asci. Asci 145.5–*146*–150 × 80–*83*–84.5 µm, Q = 1.75, quickly evanescent, inamyloid, prototunicate, subglobose, peduncle 45–65 µm long (included in the ascus measurements), with a thin wall, containing 4–8 ascospores. Ascospores widely ellipsoidal, 22–*25.5*–27 × 20–*22.5*–23.5 µm, Q = 1.1–1.15, ornamented with small ridges and tiny isolated granules, with a very thick perisporium with golden tones under the optical microscope.
  *Ecology and Distribution*: Collected only in Bulgaria, in calcareous soil, under annual species of *Helianthemum*, but maybe also associated with *Carpinus* and/or *Quercus*, in late spring and summer.
  *Additional material studied*: **Bulgaria**, Klementevo, Varna, under *Helianthemum nummularium*, *Carpinus* sp. and *Quercus* sp., 2 January 2017, *P. Bozadjiev* (BCN-myc 3604, 1923F8494); Obrochishte, Varna, under *Carpinus* sp., 8 May 2019, *M. Slavova & B. Assyov* (BCN-myc 3605*, 2330F3475).
  *Notes*: *Picoa pontica* is characterized by (1) mycelial hairs present at maturity; (2) surface with compound pyramidal warts, isolated and not very pronounced (with 4–8 edges); (3) outer layer of the peridium very thin (1–3 rows of cells), pseudoparenchymatous, made of sclerenchymatous subglobose to elongated cells, with walls up to 9.1 µm thick; (4) internal layer of the peridium plectenchymatous, intricate, made of elongated hyphae; (5) widely ellipsoidal ascospores, ornamented with small ridges and isolated tiny granules, with a very thick perisporium and golden tones under the optical microscope; (6) preference for calcareous soils; (7) probably associated with *Helianthemum* spp., maybe also *Carpinus* or *Quercus* sp. The most closely related species are *P. communis*, *P. fajardoi*, *P. montecchii*, *P. morarae*, *P. pacionii*, *P. papillata*, *P. rodensis*, *P. sphaerospora*, *P. truncata,* and *P. vazqueziae.* The first six are compared with *P. pontica* above. *Picoa rodensis* has few mycelial hairs when young, not persistent; the outer layer of the peridium has cell walls up to 13.7 µm thick (vs. 9.1 µm in *P. pontica*), and ascospores do not show a golden-colored perisporium. *Picoa sphaerospora* has irregular compound warts; the internal layer of the peridium is pseudoparenchymatous, made of gradually smaller cells, mixed with glebal tissue made of elongated hyphae arranged in parallel, not intricate; spherical ascospores, 23–24 µm diam., and has preference for neutral soils. *Picoa truncata* has darker ascomata with prominent black compound pyramidal warts with truncated apices and reddish-brown bases, as well as non-persistent mycelial hairs*;* intermediate layer of the peridium with very small subglobose cells, 7.5–15.5 × 8–15.5 µm, and ascospores ornamented with large, isolated granules. Finally, *P. vazqueziae* has black ascomata with non-persistent mycelial hairs; outer layer of the peridium with parallelepipedic cells; intermediate layer plectenchymatous and intricate, and ascospores lacking ridges and a golden perisporium.

18. ***Picoa rodensis*** A. Paz, E.J. Salvador-Fernández, C. Lavoise & J. Gómez, *sp. nov*. MB 859766. Figure 3, Figure 4 and Figure 5 and Figure 19.
  *Etymology*: “*Rodensis*” refers to the town of La Roda (Albacete, Spain), the place where the holotype was collected.
  *Type*: **Spain**, Castilla La Mancha, Albacete, La Roda, in calcareous soil, under *Cassia* sp., near *Helianthemum salicifolium*, *Tuberaria guttata*, *Rosmarinus* and *Quercus*, 10 April 2017, *E. J. Salvador-Fernández* (BCN-myc 3606 holotype, IC1041701 isotype).
  Ascomata form globose or sometimes slightly lobed stereothecia, 23–42 mm diam., dark brown to almost black, with a few mycelial hairs when young. Peridium (surface) showing pronounced compound pyramidal warts, measuring 105.5–198 µm (height) × 125–283 µm (base), with a crater-shaped center and 3–5 remarked vertical edges. Medullary excipulum (peridium section) consisting in three layers: (1) outer layer textura angularis, pseudoparenchymatous, very irregular in size, 42.5–155 µm thick, formed by 4–6 rows of cells at the apices and 2–3 rows at the bases, made of subglobose or slightly elongated cells, 14–30.5 × 10–29.5 µm, Q = 1.26, very thick-walled, up to 13.7 µm thick, reddish brown pigmented; these cells are interspersed with some isolated cylindrical bases of mycelial hairs, slightly wider at the bottom, measuring 28 × 19.5 µm on average, Q = 1.53; (2) intermediate layer 60–93 µm thick, textura angularis, pseudoparenchymatous, made of globose to polyhedrical hyaline cells, interspersed with more elongated hyphae, 9.5–14 × 8–16.5 µm (long), Q = 1.12; (3) internal layer (transition toward the gleba) textura intricate, 141–285.5 µm thick, plectenchymatous, made of increasingly smaller, elongated cells, 10–14.5 × 5.5–10 µm, Q = 1.66. Gleba composed of sterile zones with an intricate texture, formed by elongated, bifurcate hyphae, with septa measuring on average 11.5 × 3 µm, as well as disordered fertile areas with asci. Asci 52.5–*154.5*–157 × 70–*71.5*–73 µm, quickly evanescent, inamyloid, prototunicate, subglobose, peduncle 40–55 µm long (included in the ascus measurements), thin-walled, containing 4–8 ascospores. Ascospores broadly ellipsoidal, 23.5–*26.5*–28 × 21.5–*23.5*–25 µm, Q = 1.09–1.13, ornamented with abundant, pronounced small granules and isolated small ridges.
  *Ecology and Distribution*: Collected in Algeria [13,33] and Spain. Spanish collections were generally found in calcareous soils, under annual species of *Helianthemum*, and sometimes also *Cassia*, *Rosmarinus,* and *Quercus*. Occurs from late winter to late spring.
  *Additional material studied*: **Spain**, Andalucia, Granada, under *Cassia* and *Helianthemum almeriense*, 26 May 1998, *J. Gómez & P. Martínez* HB-JG-A126 (BCN-myc 3607*); Castilla y León, Zamora, Peleagonzalo, in calcareous soil, near *Cytisus scoparius* and *Quercus ilex*, 20 Jun 2015, *J. Cabero* JC100620NR.
  *Notes*: *Picoa rodensis* is characterized by (1) mycelial hairs absent at maturity; (2) surface with very pronounced, isolated compound pyramidal warts (with 3–5 edges); (3) outer layer of the peridium thin (2–6 rows of cells), pseudoparenchymatous, made of sclerenchymatous subglobose cells, with walls up to 13.7 µm thick; (4) internal peridium layer very thick, plectenchymatous, intricate, made of elongated hyphae; (5) ascospores widely ellipsoidal, ornamented with abundant, small but pronounced granules and isolated small ridges; (6) preference for calcareous soils; (7) probably associated with annual species of *Helianthemum*, maybe also with *Cassia*, *Tuberaria*, *Rosmarinus,* or *Quercus*. The most closely related species are *P. communis*, *P. fajardoi*, *P. montecchii*, *P. morarae*, *P. pacionii*, *P. papillata*, *P. pontica*, *P. sphaerospora*, *P. truncata,* and *P. vazqueziae.* The first seven species are compared with *P. rodensis* above. *Picoa sphaerospora* has spherical ascospores, 23–24 µm diam., and preference for neutral soils. *Picoa truncata* has darker ascomata covered by prominent black compound pyramidal warts with truncate apices; outer and intermediate peridium layers made of smaller subglobose cells; and ascospores ornamented with large, isolated granules. Finally, *P. vazqueziae* has black ascomata covered with very prominent warts; outer layer of the peridium made of parallelepipedic to elongated cells, and ascospores ornamented with small, isolated granules and without isolated ridges.

19. ***Picoa sphaerospora*** A. Paz, J. Gómez, A.D. Exposito-Gómez & C. Lavoise, *sp. nov*. MB 859767. Figure 3, Figure 4 and Figure 5 and Figure 20.
  *Etymology*: From the Ancient Greek σφαῖρα (*sphaîra*) meaning sphere or globe, and σπορά (*sporá*) meaning seed, in relation to the globose ascospores.
  *Type*: **Spain**, Andalucia, Córdoba, Priego de Córdoba, under *Cistus albidus*, 18 March 1991, *J. Gómez* (BCN-myc 3608 holotype, IC18039115 isotype).
  Ascomata form subglobose stereothecia, up to 28 mm diam., reddish brown to dark brown or almost black in old age, initially wrapped with reddish brown mycelial hairs that disappear with age or handling. Peridium (surface) with prominent, irregular compound warts, measuring 252.5–364.5 µm (height) × 262–573 µm (base), with 3–4 edges. Medullary excipulum (peridium section) arranged in three layers: (1) external layer 49–102.5 µm thick, textura angularis, pseudoparenchymatous, made of subglobose to polyhedrical sclerenchymatous cells, very irregular in shape and size, measuring 23.5 × 26 µm on average, Q = 0.97, with walls up to 5.7 µm thick, reddish brown pigmented, in rows of 2–3 at the base of the warts and 3–6 at the apices; (2) intermediate layer 37.5–110 µm thick, textura angularis, pseudoparenchymatous, made of polyhedrical or puzzle-like, hyaline, sclerenchymatous cells, 12.5–26 × 10–34.5 µm, Q = 0.89, with increasingly thinner walls up to 1.8 µm thick; (3) internal layer (transition toward the gleba) textura angularis, pseudoparenchymatous, made of gradually smaller cells, 5.5–10.5 × 6.5–15 µm, Q = 0.95, mixed with glebal tissue made of elongated hyphae arranged in parallel. Gleba composed of sterile zones with an intricate texture, made of elongated, bifurcate hyphae, with septa of 10.5 × 3.5 µm on average, as well as disordered fertile areas, with asci. Asci 140–*141*–143.5 × 65–*65.5*–67 µm, quickly evanescent, inamyloid, prototunicate, subglobose, peduncle 55–75 µm long (included in the ascus measurements), with a thin and simple wall, containing (3–)4–7(–8) ascospores. Ascospores spherical, 23–24 µm diam., Q = 1, ornamented with minute but pronounced isolated granules.
  *Ecology and Distribution*: Apparently abundant in southern Spain (Andalusia), in the provinces of Córdoba and Jaén, and also collected in Algeria, El-Bayadh, in sandy soil with a neutral pH, from mid-February to the end of May.
  *Additional material studied*: **Spain**, Andalucia, Cordoba, Priego de Córdoba, Paraje Las Cabrerizas de Albayate, Campillo slope, under *Helianthemum* sp., 10 April 1993, *coll. B. Moreno-Arroyo* (BM314*); Jaén, Úbeda, Madre de Dios del Campo, ecological olive grove with sandy soil populated with *Helianthemum* sp., neutral soil, in small groups of 4 to 5 specimens, 780 m, 2 March 2020, *A. D. Expósito-Gómez* (BCN-myc 3609, IC02032001); ibid. 18 February 2021 (IC18022101); ibid. 9 March 2022 (IC09032201).
  *Notes*: *Picoa sphaerospora* is characterized by having (1) spherical ascospores 23–24 µm diam., ornamented with minute but pronounced isolated granules; (2) surface with prominent, irregular compound warts with 3–4 edges; (3) mycelial hairs absent at maturity; (4) outer layer of the peridium pseudoparenchymatous, made of subglobose to polyhedrical sclerenchymatous cells; (5) inner layer of the peridium pseudoparenchymatous, made of puzzle-shaped cells, interspersed with elongated hyphae in parallel; (6) preference for neutral soils; (7) probably associated with annual species of *Helianthemum* sp. The most closely related species are *P. communis*, *P. fajardoi*, *P. montecchii*, *P. morarae*, *P. pacionii*, *P. papillata*, *P. pontica*, *P. rodensis*, *P. truncata,* and *P. vazqueziae.* The first eight species are compared with *P. sphaerospora* above. *Picoa truncata* has darker ascomata, covered by prominent black, compound pyramidal warts with truncated apices*;* internal layer of the peridium plectenchymatous, intricate; broadly ellipsoidal ascospores, 25–27.5 × 23–24 µm, and preference for calcareous soils. Finally, *P. vazqueziae* has black ascomata, covered with very prominent warts; outer layer of the peridium made of parallelepipedic to elongated cells; and ascospores widely ellipsoidal, 23–27.5 × 20.5–24 µm, and preference for calcareous soils.

20. ***Picoa truncata*** A. Paz, C. Lavoise, G. Konstantinidis, J.A. Martínez & J.M. Martínez, *sp. nov*. MB 859768. Figure 3, Figure 4 and Figure 5 and Figure 21.
  *Etymology*: From the Latin verb *truncare*, to truncate, in reference to the truncated apices of the warts on the surface of ascomata.
  *Type*: **Spain**, Castilla La Mancha, Cuenca, Belinchón, in calcareous soil, with annual *Helianthemum* sp. and *Pinus halepensis*, 4 April 2017, *J. A. Martínez* & *J. M. Martinez* (BCN-myc 3610 holotype, IC4041703 isotype).
  Ascomata form globose stereothecia, up to 38 mm diam., covered with black warts with a reddish-brown base, as well as mycelial hairs when young. Peridium (surface), with prominent compound pyramidal warts generally showing 2–4 edges and a truncated apex, measuring 257–412.5 µm (high) × 313.5–520.5 µm (base). Medullary excipulum (peridium section) arranged in three layers: (1) outer layer about 48.5–65 µm thick, formed by 3–4 (–5) rows of cells at the apices and 2–3 at the bases of the warts, textura angularis, pseudoparenchymatous, made of small subglobose sclerenchymatous cells, 12–28.5 × 11.5–24.5 µm, Q = 1.08, with walls up to 6.8 µm thick, dark brown pigmented; these cells are interspersed with the bases of many conical mycelial hairs, measuring 19.5 × 14.5 µm on average, hairs septate, measuring 120 × 8 µm on average; (2) intermediate layer 102.5–146 µm thick, textura angularis, made of very small, hyaline, sclerenchymatous globose to polyhedric cells, 7.5–15.5 × 8–15.5 µm, Q = 0.99; (3) internal layer (transition toward the gleba) 327.5–498 µm thick, textura intricate, composed by a plectenchymatous structure made of increasingly elongated and intrincate cells, measuring 11–22 × 5–8 µm, Q = 0.39. Gleba containing sterile zones with an intricate texture, made of elongated and bifurcate hyphae, with septa measuring 11 × 4.5 µm on average, as well as disordered fertile areas with asci. Asci 162.5–*163*–166.5 × 66.5–*68*–70.5 µm on average, quickly evanescent, inamyloid, prototunicate, subglobose, peduncle 50–65 µm long (included in the ascus measurements), with a thin and simple wall, containing 4–8 ascospores. Ascospores broadly ellipsoidal, 25–*26.5*–27.5 × 23–*23.5*–24 µm, Q = 1.08–1.12, ornamented with large isolated granules.
  *Ecology and Distribution*: Found so far in Algeria [13,33], Greece, Italy, and Spain, in nutrient-poor calcareous soils, maybe associated with *Pinus halepensis* and/or *Helianthemum* sp., in spring (March-April).
  *Additional material studied*: **Greece**, West Macedonia, Kozani, Petrana, near *Helianthemum* sp., 30 March 2013, *I. Karagkiozis* GK6582*.
  *Notes*: *Picoa truncata* is characterized by (1) mycelial hairs absent at maturity; (2) surface with compound pyramidal warts, showing 2–4 edges, truncated and with a reddish base; (3) outer layer of the peridium thick (2–5 rows of cells), pseudoparenchymatous, made of subglobose, sclerenchymatous cells, with walls up to 6.8 µm thick; (4) intermediate layer of the peridium pseudoparenchymatous, made of very small globose to polyhedric cells, 11.5 × 12 µm on average; (5) ascospores broadly ellipsoidal, ornamented with large isolated granules; (6) preference for calcareous soils; (7) probably associated with *Helianthemum* sp. or maybe *Pinus halepensis*. The most closely related species are *P. communis*, *P. fajardoi*, *P. montecchii*, *P. morarae*, *P. pacionii*, *P. papillata*, *P. pontica*, *P. rodensis*, *P. sphaerospora,* and *P. vazqueziae.* The first nine species are compared with *P. truncata* above. *Picoa vazqueziae* produces ascomata covered by very prominent warts with acute, not truncated, apices; outer layer of the peridium made of parallelepipedic to elongated cells; and ascospores ornamented with tiny, isolated granules.

21. ***Picoa vazqueziae*** A. Paz, J. Cabero & C. Lavoise, *sp. nov*. MB 859769. Figure 3, Figure 4 and Figure 5 and Figure 22.
  *Etymology*: Dedicated to Begoña Vázquez Delgado, a great Spanish collector of hypogeous fungi.
  *Type*: **Spain,** Castilla y León, Zamora, Peleagonzalo, in calcareous soil, in a forest clearing near *Quercus ilex* subsp. *ballota* and *Tuberaria* sp., 6 December 2013, *J. Cabero* (BCN-myc 3611 holotype, JC131206NR isotype, IC14091543 isotype).
  Ascomata form globose to lobed stereothecia, up to 34 mm diam., black, very compact, resembling *Tuber aestivum*, but with a few mycelial hairs when young. Peridium (surface), with very large compound pyramidal warts, with 2–5 edges, measuring 406–567 µm (height) × 1029–1338 µm (width). Medullary excipulum (peridium section) composed of three layers: (1) outer layer textura angularis, pseudoparenchymatous, 317.5–549 µm thick, formed by 8–10 (–12) rows of cells at the apices and 6–8 rows at the bases, made of parallelepipedic to elongated cells, vertically parallel, 14–33 × 11.5–19 µm, Q = 0.83, dark reddish brown pigmented, with walls up to 9.5 µm thick, interspersed with the cylindrical bases of some isolated mycelial hairs, measuring 29 × 13 µm on average; (2) intermediate layer texture intricate, plectenchymatous, 65.5–101.5 µm thick, made of very intertwined elongate cells, 9–18.5 × 6–15 µm, Q = 1.36, mixed with some small globose to polyhedric cells; (3) internal layer (transition toward the gleba) texture intricate, plectenchymatous, 185–321.5 µm thick, made of cells measuring 12–24.5 × 5.5–10 µm, Q = 2.34. Gleba composed of sterile zones with an intricate texture, made of elongated, bifurcate hyphae, with septa measuring 10 × 3.5 µm on average, as well as disordered fertile areas with asci. Asci 125–*125.5*–126.5 × 65–*66.5*–69.5 µm, Q = 1.28, quickly evanescent, inamyloid, prototunicate, subglobose, peduncle 40–55 µm long (included in the ascus measurements), thin-walled, containing 4–8 ascospores. Ascospores widely ellipsoidal, 23–*25.5*–27.5 × 20.5–*22.5*–24 µm, Q = 1.1–1.15, ornamented with tiny, isolated granules.
  *Ecology and Distribution*: Collected so far in Spain and Iran [32], apparently in calcareous soil, in association with annual species of *Helianthemum* and *Tuberaria* sp., sometimes near *Quercus*, in summer and early autumn.
  *Additional material studied*: **Spain**, Castilla y León, Zamora, Peleagonzalo, calcareous soil in forest clearing near *Quercus ilex* subsp. *ballota* and *Tuberaria* sp., 18 January 2018, *B. Vázquez* (BCN-myc 3612, IC18061813); Ibid. 21 Oct. 2021 (IC21102101*); Castilla-La Mancha, Albacete, Casas de Lázaro, 14 May 2004, *A. Rodríguez* (AH:39035*); Guadalajara, Membrillera, with *Helianthemum* sp., 6 Nov 2010, M. Á. Sánz (AH:39139*).
  *Notes*: *Picoa vazqueziae* is characterized by (1) black-colored ascomata and absent or very scarce mycelial hairs when young; (2) surface with very large, compound, pyramidal warts, with 2–5 edges; (3) outer layer of the peridium very thick (6–10 rows), pseudoparenchymatous, made of elongated parallelpipedic cells, vertically parallel, with walls up to 9.5 µm thick; (4) internal layer of the peridium plectenchymatous, intricate, with hyphae larger than those of the intermediate layer; (5) ascospores widely ellipsoidal, ornamented with tiny isolated granules; (6) preference for calcareous soil; (7) associated with annual species of *Helianthemum* and maybe also *Tuberaria.* The most closely related species are *P. communis*, *P. fajardoi*, *P. montecchii*, *P. morarae*, *P. pacionii*, *P. papillata*, *P. pontica*, *P. rodensis*, *P. truncata,* and *P. sphaerospora* (see above).

22. ***Picoa*** sect. ***Microsporae*** P. Alvarado. A. Paz & Van Vooren, *sect. nov*. MB 859774. Figure 3, Figure 4 and Figure 5 and Figure 23.
  *Etymology*: Named after the type, *P. microspora.*
  *Type*: *Picoa microspora* A. Paz, C. Lavoise & G. Pacioni.
  The section *Microsporae* is characterized by species with the following characteristics: (1) reddish-brown ascomata, (2) outer surface covered with pyramidal warts, (3) outer peridium layer made of sclerenchymatous cells, with thin walls, up to 3.1 µm thick, (4) ascospores 21.5 × 18.5 µm on average, decorated with very thick granules and roughness, (5) linked to *Helianthemun* spp. on calcareous soil. It contains a single species, *P. microspora.*

23. ***Picoa microspora*** A. Paz, C. Lavoise & G. Pacioni, *sp. nov*. MB 859770. Figure 3, Figure 4 and Figure 5 and Figure 23.
  *Etymology*: From the ancient Greek μικρός (*mikros*) meaning small and σπορά (*sporá*) spora meaning seed, referring to the size of ascospores, the smallest of the known species of *Picoa*.
  *Type*: **Egypt**, Sidi Barrani, 28 February 1992, *G. Pacioni* & *El Kholy* (BCN-myc 3614 holotype, AQUI7051 isotype).
  Ascomata form subglobose stereothecia, 23 mm diam. on average, intensely reddish brown, slightly darkening when mature, with mycelial hairs present when young, sparse when mature. Peridium (surface) with irregular pyramidal warts, measuring 81–327.5 µm (height) × 235.5–578.5 µm (base), warts sometimes compound, with 2–3 edges, or simple. Medullary excipulum (peridium section) arranged in three layers: (1) outer layer very irregular in size, 22–53 µm thick, textura angularis, pseudoparenchymatous, made of subglobose to elongated sclerenchymatous cells, typically arranged in 2–3 rows at the apices of the warts and 1 row at the bases, 19.5–43.5 × 16.5–35.5 µm, Q = 1.11, slightly brown pigmented, walls up to 3.1 µm thick; the outer layer can be exceptionally formed of 4–6 rows of cells, 136.5–159.5 µm thick at the apices of warts where they are more irregular and sharp; (2) intermediate layer 39.5–98.5 µm thick, textura angularis, pseudoparenchymatous, made of hyaline, polyhedric to irregular sclerenchymatous cells, 11.5–27.5 × 13–31.5 µm, Q = 1.08, with walls up to 2.9 µm thick, interspersed with septate hyphae; (3) inner layer (transition toward the gleba) 136–267.5 µm thick, textura angularis, pseudoparenchymatous, made of irregularly polyhedric or puzzle-like cells, 4–12.5 × 6.5–17.5 µm, Q = 1.62, interspersed with bifurcated interwoven hyphae from the glebal tissue. Gleba composed of sterile zones with an intricate texture, made of elongated, bifurcate hyphae, with septa measuring 11.5 × 4 µm on average, as well as disordered fertile cells, with asci. Asci 142.5–*143*–145.5 × 66.5–*68*–70.5 µm, quickly evanescent, inamyloid, prototunicate, subglobose, peduncle 55–75 µm long (included in the ascus measurements), with a thin and simple wall, containing 4–8 ascospores. Ascospores broadly ellipsoidal, 20.5–*21.5*–22.5 × 18–*18.5*–20 µm, Q = 1.12–1.15, ornamented with isolated, thick granules, scattered on the rough spore surface.
  *Ecology and Distribution*: So far collected in Cyprus, Egypt, and Tunisia [13,33] in sandy, calcareous soils, in association with *Helianthemum*, in late winter and spring.
  *Additional material studied*: **Cyprus**, Mesaoria, near *Helianthemum* sp., 1 April 2009, *Y. Yangou* (GK3737*).
  *Notes*: *Picoa microspora* is characterized by (1) sparse mycelial hairs at maturity; (2) surface with mixed pyramidal warts (with 2–3 edges); (3) outer layer of the peridium irregular (1–6 rows of cells), pseudoparenchymatous, made of sclerenchymatous cells, with thin walls up to 3.1 µm thick; (4) inner peridium layer pseudoparenchymatous, made of polyhedric cells and interspersed elongated forked hyphae; (5) ascospores very small, less than 22.5 µm in length (the smallest in the genus *Picoa*), widely ellipsoidal, ornamented with coarse granules, scattered over the rough spore surface; (6) preference for sandy, calcareous soils; (7) probably associated with perennial species of *Helianthemum*; (8) apparently restricted to the Levant (Cyprus) and the African shore of the Mediterranean basin (Egypt, Tunisia). The size of the ascospores differentiates it from the other species, only *P. puentei* has more or less similar small ascospores, 22–23.5 × 19.5–21 µm µm (slightly wider than *P. microspora*), but *P. puentei* has an ectal excipulum covered with very irregular isolated granules and a plectenchymatous inner layer, with cells horizontally parallel toward the gleba, not intricate.

24. ***Picoa*** sect. ***Puenteorum*** P. Alvarado, A. Paz & Van Vooren, *sect. nov*. MB 859775. Figure 3, Figure 4 and Figure 5 and Figure 24.
  *Etymology*: Named after the type, *P. puentei.*
  *Type*: *Picoa puentei* A. Paz, F. García & C. Lavoise.
  The section *Puenteorum* is characterized by species with the following characteristics: (1) dark reddish-brown ascomata, covered by granules (without halo); (2) outer layer of the peridium made of sclerenchymatous cells; (3) ascospores 22.5 × 20 µm on average, ornamented with minute granules that join together forming small irregular ridges; (4) probably associated with Cistaceae (*Helianthemum*, *Cistus*, *Tuberaria*), with *Pinus* or *Quercus* present, on calcareous soil. It contains a single species, *P. puentei.*

25. ***Picoa puentei*** A. Paz, F. García & C. Lavoise, *sp. nov*. MB 859771. Figure 3, Figure 4 and Figure 5 and Figure 24.
  *Etymology*: Dedicated to Alberto Pérez Puente for his work in disseminating mycology as president of the Cantabrian Mycological Society during 32 years.
  *Type*: **Spain**, Castilla y León, Segovia, in soil near *Quercus pyrenaica* and *Helianthemum* spp., 12 May 2002, *F. García* (BCN-myc 3615 holotype, IC12050266 isotype).
  Ascomata form globose stereothecia, 17–26 mm diam., intensely reddish brown to dark reddish brown (at maturity), without persistent mycelial hairs. Intense smell. Peridium (surface) covered with very irregular isolated granules, measuring 98.5–158.5 µm (height) × 241–419 µm (base). Medullary excipulum (peridium section) arranged in three layers: (1) outer layer 22–52.5 µm thick, formed by 2–3 rows of cells at the apices and 1–2 rows at the bases, textura angularis, pseudoparenchymatous, made of subglobose or slightly elongated sclerenchymatous cells, 10–24 × 9.5–19.5 µm, Q = 1.25, walls up to 3.5 µm thick, reddish brown pigmented; (2) intermediate layer 70.5–120 µm thick, textura angularis, made of globose to polyhedric sclerenchymatous, hyaline cells, 9–15.5 ×11–21 µm, Q = 0.82; (3) inner layer (transition toward the gleba) 159.5–256 µm thick, plectenchymatous, made of increasingly smaller, elongated, cylindrical cells, horizontally parallel toward the gleba, 5.5–10.5 × 9.5–21 µm, Q = 0.54. Gleba composed of sterile zones with an intricate texture, made of elongated, bifurcate hyphae, with septa measuring 13.5 × 4.5 µm on average, as well as disordered fertile cells with asci. Asci 144–*145.5*–150.5 × 68.5–*70.5*–74 µm, Q = 2.06, quickly evanescent, inamyloid, looking light brown when mature, prototunicate, subglobose to broadly ellipsoidal, peduncle 50–65 µm long (included in the ascus measurements), thin-walled; containing 6–8 ascospores. Ascospores broadly ellipsoidal, 22–*22.5*–23.5 × 19.5–*20*–21 µm, Q = 1.11–1.13, ornamented with very fine minute granules which join at the bases to form small, very irregular ridges, with a thick perisporium, golden-orange color.
  *Ecology and Distribution*: So far collected only in central Spain (Castilla y León, provinces of Burgos, Segovia and Valladolid), in association with *Helianthemum* and maybe also other Cistaceae (*Tuberaria*, *Cistus*), sometimes in the presence of *Quercus* or *Pinus*. It seems to prefer sandy calcareous soils in spring.
  *Additional material studied*: **Spain**, Castilla y León, Burgos, Llanos de la Bureba, in sandy soil with *Cistus salviifolius*, *Tuberaria guttata* and *Helianthemum* sp., 19 May 2007, F. Sáinz (AH:39246*). Valladolid, Villanueva del Duero, in sandy soil, in a pine forest with *Helianthemum* sp., 16 January 1994, *A. García-Blanco*, *M. Sanz* & *J. B del Val* (BCN-myc 3616*, AVM1005).
  *Notes*: *Picoa puentei* is characterized by (1) sparse mycelial hairs when young; (2) surface covered with large irregular isolated granules, without halo; (3) outer layer of the peridium very thin (1–3 rows of cells), pseudoparenchymatous, made of subglobose or slightly elongated sclerenchymatous cells, with very thin walls, up to 3.5 µm thick; (4) intermediate layer of the peridium pseudoparenchymatous, made of small globose to polyhedric sclerenchymatous cells, measuring 12 × 16 µm on average, (5) widely ellipsoidal ascospores, ornamented with minute granules joining at their bases to form small, very irregular ridges, with a thick perisporium, in golden-orange tones; (6) preference for sandy calcareous soils; (7) probably associated with *Helianthemum*, and maybe also *Quercus*, *Pinus*, *Cistus* and/or *Tuberaria*. See also notes under *P. microspora* above.

**Figure 24:**
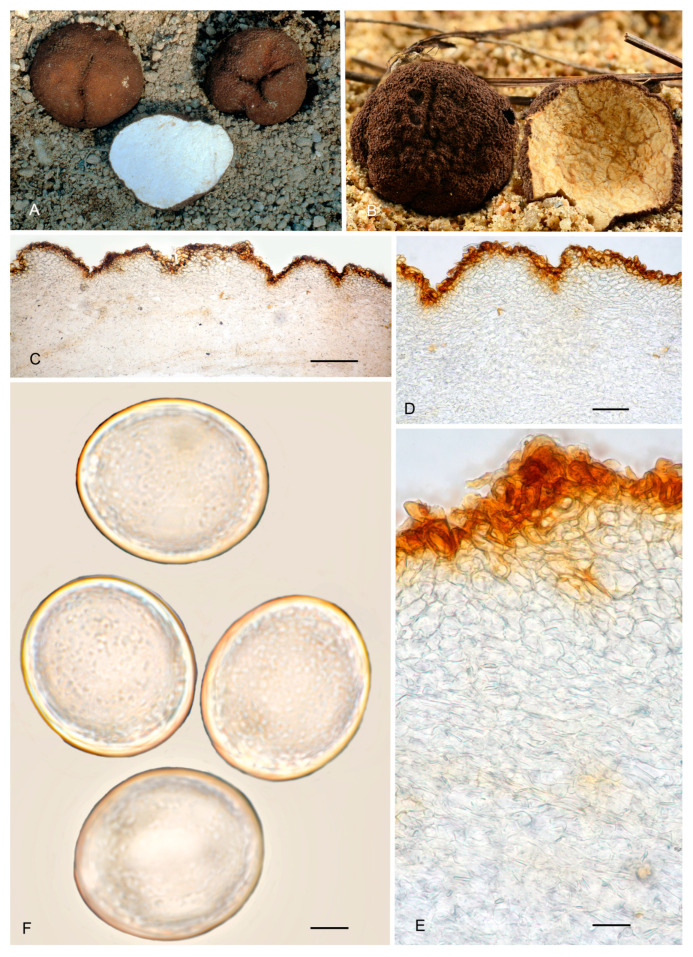
*Picoa puentei* BCN-myc 3615 (holotype): (**A**) ascomata; (**B**) surface detail; (**C**) section of peridium and gleba; (**D**,**E**) medullary excipulum with pseudoparenchymatous structure, made of subglobose or slightly elongated sclerenchymatous cells; (**F**) ascospores. Scale bars: (**C**) = 2 mm; (**D**) = 40 µm; (**E**) = 20 µm; (**F**) = 5 µm. Photos: (**A**) = F. García (from fresh material); (**B**) = A. Paz (from dry material); (**C**–**F**) = A. Paz (from rehydrated mounts).

## 4. Discussion

The present work proposes several taxonomic novelties based on an updated phylogeny of the genus *Picoa* and related lineages. The genus *Sepultaria* is considered independent from *Geopora* s. str., and *Picoa* is organized into five distinct sections and at least 19 species.

The genus *Geopora* was published by Harkness [34] based on a hypogeous species, *G. cooperi*. The holotype, collected in California (USA), had the appearance of some species of *Hydnotrya* Berk. & Broome but with “oblong and smooth” spores. *Geopora* was described as “Subterranean. Integument woolly, continuous with the trama. Hymenium convolute. Asci cylindrical. Sporidia hyaline, oblong, smooth.” Burdsall [35] highlighted that the ascospores of *G. cooperi* form a cloud when a fresh specimen is broken. He also observed a functional operculum in the ascus. This structure, which allows for the forcible discharge of ascospores, is found in almost all Pezizales but not in the former order Tuberales, where the genus *Geopora* was previously classified. Three years later, Burdsall [36] merged *Sepultaria* and *Geopora* (including some species of *Hydnocystis* Tul. & C. Tul.) on the basis of shared morphological characteristics. The name *Sepultaria* was first used by Cooke [37] as a subgenus of *Peziza* with the following description: “*Cupula carnosa*, *sessilis*, *extus pilis coloratis vestita*.” Cooke’s definition encompassed heterogeneous species, but those with “*sporidiis ellipticis*” were later integrated in Boudier’s restricted definition of the genus *Sepultaria* [38,39]. This circumscription corresponds to a group of species first developing below the ground but breaking through it later (semi-hypogeous), with a hairy external surface, and producing hyaline ascospores containing one or two large oil bodies accompanied by small droplets. The name *Geopora* was retained and amended by Burdsall [36] because it has nomenclatural priority over *Sepultaria*. This widened concept of *Geopora* was followed by subsequent mycologists such as Korf [40], Torre [41], Ahmad [42], Schumacher [43], Yao and Spooner [44], Dissing et al. [45], Medardi [46], Dougoud [47], Tamm et al. [48], etc., with the exception of Dennis [49] and Breitenbach and Kränzlin [50], who preferred the name *Sepultaria*.

The first comprehensive phylogeny of *Geopora* built on the basis of ITS rDNA sequences was published by Tamm et al. [48]. Kaounas et al. [51] found that two different candidate clades of *Geopora clausa*, a semi-hypogeous ptychothecial species, were not directly related to the type species of *Geopora*, *G. cooperi*, but nested among epigeous apothecial taxa of *Geopora* (including the type of *Sepultaria*). As a result, these authors hypothesized that *Geopora* s. str. should not be considered a synonym of *Sepultaria*, a point of view followed later by Guevara-Guerrero et al. [52] and Van Vooren [53]. In the present work, this hypothesis was further confirmed with an extended dataset including sequences of rDNA and protein-coding genes of *Geopora*, *Sepultaria*, and the closely related genera *Picoa* and *Terracavicola*. Beyond their genetic profile, the main difference between *Geopora* and *Sepultaria* is probably the rather brittle flesh of *Geopora*, caused by a rather thin and poorly differentiated ectal excipulum. The main features of these four related genera containing sequestrate species are summarized below.

***Sepultaria***. Type: *Peziza sepulta* Fr. 1851, designated by Boudier [38]. Ascomata isolated or gregarious, mainly apothecial, but sometimes ptychothecial. Apothecia first globose to subglobose, opening through a somital hole, then spreading out; ptychothecia subglobose or potato-like, often convoluted, hollow, with a single chamber or with convoluted chambers. External surface often verrucose, covered with a more or less abundant brown tomentum. Hymenium distinct, beige- to gray-colored. Asci cylindrical, arising from croziers, persistent, inamyloid, containing eight uniseriate spores. Sclerenchymatous cells present. Ascospores widely ellipsoid to ellipsoid, sometimes tapered at the ends, uni- or biguttulate, accompanied by small droplets, uninucleate, hyaline, thick-walled, smooth. Excipulum distinctly bilayered, with a medullary excipulum made of textura intricata with hyaline hyphae and an ectal excipulum made of textura globulosa/angularis. Peridium made of angular brown cells, sometimes organised in small pyramidal aggregates. Hairs superficial, yellow-brown to brown, thick-walled, flexuous, obtuse. Asexual morph unknown. Trophic status: ectomycorrhizal.

***Geopora***. Type: *Geopora cooperi* Harkn. 1885, designated by Harkness [34]. Ascomata isolated or gregarious, mainly ptychothecial, but sometimes apothecial. Apothecia first globose, opening through a somital hole, then spreading out; ptychothecia subglobose or potato-like, convoluted, not chambered. External surface often verrucose, covered with a more or less abundant brown tomentum. Hymenium distinct, beige to light grey-coloured. Asci cylindrical, arising from croziers, persistent, inamyloid, containing eight uniseriate spores. Paraphyses present. Ascospores widely ellipsoid to ellipsoid, sometimes tapered at the ends, uni- or biguttulate, accompanied by small droplets, uninucleate, hyaline, thick-walled, smooth or finely warted. Excipulum more or less bilayered, with a medullary excipulum made of textura intricata with hyaline hyphae and a thin ectal excipulum made of subglobose, clavate, or elongated angular cells oriented in parallel with the hymenium. Peridium made of angular brown cells, sometimes organized in small pyramidal aggregates. Hairs superficial, yellow-brown to brown, thick-walled, flexuous, obtuse. Asexual morph unknown. Trophic status: ectomycorrhizal.

***Picoa***. Type: *Picoa juniperi* Vittad. 1831, designated by Vittadini [1]. Ascomata solitary or gregarious, stereothecial. Stereothecia globose to subglobose, café-au-lait, deep reddish brown, brown, dark brown, or black, with or without permanent mycelial hairs. Ectal excipulum (peridium surface) with granules, papillae, or warts. Medullary excipulum (peridium section) arranged in three layers: (1) outer pseudoparenchymatous layer with or without parallelepiped cells, (2) intermediate pseudoparenchymatous layer with or without parallelepiped or plectenchymatous cells, (3) inner layer mostly intricate or parallel-plectenchymatous or pseudoparenchymatous. Hymenium distinct. Asci cylindrical to subglobose, with a peduncle, evanescent, inamyloid, containing four to eight uniseriate spores. Paraphyses present. Ascospores spherical, broadly ellipsoidal or ellipsoidal, hyaline or with a golden perisporium, thick-walled, ornamented with granules, rugosities or ridges which join at their bases forming semi-reticles.

***Terracavicola***. Type: *Terracavicola echinospora* A. Grupe, Kraisit., Guevara & M.E. Sm. 2019, designated by Grupe II et al. [22]. Ascomata hypogeous, truffle-like, creamy-tan to light brown, with a roughened peridium, covered by copious brown hairs (setae) that are branched and microscopically textured; presence of a weak epithecium which covers the hymenium and prevents active discharge of the weakly spinose ascospores.

Some taxonomic changes are proposed below to combine several species of *Geopora* into *Sepultaria*, although both genera need a thorough revision to clarify the status of many other taxa.

***Sepultaria ahmadii*** (Saba, T. Ashraf, Khalid & Pfister) P. Alvarado, Van Vooren & A. Paz, *comb. nov.* MB 859740.

Basionym: *Geopora ahmadii* Saba, T. Ashraf, Khalid & Pfister, in Saba *& al*., *Mycotaxon*, 134 (2): 382 (2019).

***Sepultaria asiatica*** (S. Jabeen, Asghar, Niazi & Khalid) P. Alvarado, Van Vooren & A. Paz, *comb. nov.* MB 859741.

Basionym: *Geopora asiatica* S. Jabeen, Asghar, Niazi & Khalid, *Nova Hedwigia*, 114 (3–4): 394 (2022).

***Sepultaria cercocarpi*** (D. Southw. & J.L. Frank) P. Alvarado, Van Vooren & A. Paz, *comb. nov.* MB 859742.

Basionym: *Geopora cercocarpi* D. Southw. & J.L. Frank, *Mycologia*, 103 (6): 1196 (2011).

***Sepultaria clausa*** (Tul. & C. Tul.) P. Alvarado, Van Vooren & A. Paz, *comb. nov.* MB 859743.

Basionym: *Genea clausa* Tul. & C. Tul., *G. bot. ital*., 2 (1): 59 (1844).

***Sepultaria pinyonensis*** (L. Flores-Rentería & C.A. Gehring) P. Alvarado, Van Vooren & A. Paz, *comb. nov.* MB 859744.

Basionym: *Geopora pinyonensis* L. Flores-Rentería & C.A. Gehring, *Mycologia*, 106 (3): 556 (2014).

***Sepultaria ramila*** (S. Jamali & M. Sheibani) P. Alvarado, Van Vooren & A. Paz, *comb. nov.* MB 859745.

Basionym: *Geopora ramila* S. Jamali & M. Sheibani, *Phytotaxa*, 475 (1): 34 (2019).

The division of *Picoa* into the sections proposed herein is consistent with the phylogenetic structure observed before by Sbissi et al. [33], Kaounas et al. [51], and Guevara-Guerrero et al. [52]. Alvarado [54] explicitly identified five lineages using ITS rDNA data, and later, Zitouni-Haouar et al. [13] found a sixth clade using ITS, LSU, and RPB2 sequences. These works found that the phylogenetic structure observed in *Picoa* is related to specific ecological features, such as plant hosts and geographical distribution, while the classical features, such as spore ornaments and macroscopic morphology, did not allow the authors to fully discriminate the six main clades identified. These lineages were not formally described in these works because the phylogenetic species observed could not be named unambiguously due to insufficient data. The type of *Picoa*, *P. juniperi*, was originally found in autumn and winter, under *Juniperus*, in northern Italy [1], but no samples from this region and season could be analyzed by Zitouni-Haouar et al. [13]. The only samples found under *Juniperus* sp. (AH 39001) or during the winter season (AH 38893 and AH 39139) belonged to the most widespread clade in Europe (clade VI in [13]; here, sect. *Communes*).

In the present work, a suitable epitype for *P. juniperi* is designated (BCN-myc 3617), found in Italy, Bologna, Guzzano Pianoro, in sandy soil, fruiting in late autumn under *Quercus pubescens*, *Pinus* sp., and *Juniperus communis*. Phylogenetic analyses show that it is nested in clade IV in Zitouni-Haouar et al. [13], which is here named sect. *Juniperi*. The two known species belonging to this section, *P. juniperi* and *P. macrospora*, occur in the European shore of the Mediterranean basin, typically associated with *Quercus* trees, with or without *Juniperus* spp. A third lineage (represented by sample AH 39285, from L’Aquila, Italy) seems subsignificantly related to *P. juniperi* (PP 0.81, BP 68) in the analyses based on ITS rDNA (Figure 2) but not in the analyses based on ITS, LSU, and RPB2 data (Figure 1). It is provisionally named *Picoa* aff. *juniperi*, but it probably represents a distinct species to be described if other samples are found. Interestingly, all three lineages of sect. *Juniperi* (*P. juniperi*, *P. macrospora,* and AH 39285) come from samples found in summer–autumn (June–December), a feature rarely observed in the other sections (typically found in late winter to spring, January–May). The correlation between geography (Europe), putative hosts (*Quercus*), and fruiting season (summer-autumn) in the entire clade seems to reinforce the hypothesis that ecology was one of the driving factors behind the evolutionary history of *Picoa*.

The other main clades of *Picoa* are characterized by a mixture of ecological, geographical, and morphological traits. Section *Lefebvreorum* is present in Europe and Africa (including the Canary Islands) and the Middle East (Israel, Iraq, and Saudi Arabia). Its members are associated with perennial Cistaceae species of *Helianthemum* (*P. lefebvrei*; *P. llobregatina*) and *Cistus* (*P. cordubensis*; *P. pubescens*). The highly diverse lineage of *P. lefebvrei* might actually include several different independent species, with four major clades observed in the analysis of ITS rDNA and ITS-LSU-RPB2 (Figure 1 and Figure 2). While the phylogenetic structure of sect. *Lefebvreorum* seems similar to that observed in the other sections of *Picoa* (where the different clades are considered distinct species), the number of samples from this grex available in the present study was too limited (only six samples of Lefebvrei-IV and one of Lefebvrei-III) to draw reliable taxonomic conclusions. While the geographical distribution and plant hosts of these lineages seem very similar, additional samples are necessary to look for putative diagnostic morphological features. The available name *Terfezia schweinfurthii* Henn., previously considered a synonym of *P. lefebvrei*, should be taken into account, as it might actually refer to a different clade.

Species of sect. *Microsporae* are also present in Africa and the Mediterranean Levant (Egypt; Cyprus), but they are apparently much less frequent than species of sect. *Lefebvreorum*. They seem associated with perennial species of *Helianthemum* as well, although additional samples are necessary to produce reliable hypotheses about their ecology. Until now, species of the section *Puenteorum* have only been found in central Spain (Burgos, Segovia, and Valladolid), a formerly endorreic sedimentary region around the river Duero, formed during the Neogene (Miocene–Pliocene). The phylogenetic analyses conducted in the present work (Figure 1 and Figure 2) suggest that this is the oldest extant lineage of *Picoa*, pointing to the Iberian Peninsula as a putative ancestral homeland. The plant hosts in this section are still uncertain, as the only three known samples were found near several Cistaceae genera (*Helianthemum*, *Cistus*, and *Tuberaria*) and trees (*Pinus*, *Quercus*). The only common genus present in association with all samples is *Helianthemum*, so this is the most probable host candidate.

Most European samples of *Picoa* belong to sect. *Communes*, but this clade is also present in Africa (Algeria, Egypt, and Tunisia) and the Middle East (Turkey, Iraq, and Iran). Most collections occur near annual species of *Helianthemum*, frequently in the presence of other putative hosts of the family Cistaceae and/or Mediterranean trees (*Quercus*; *Pinus*). Section *Communes* is here described with ten distinct taxa, but it could include five other species on the basis of ITS rDNA data available (Figure 2). There seems to be a high intraspecific variability, mainly in the ITS rDNA, but also in LSU and RPB2 sequences, which produces long branches and significantly monophyletic lineages inside some of these species. Some of them, i.e., *P. pacionii*, exhibit a remarkably low (92%) overall intraspecific similarity of ITS sequences. A closer look at them reveals two main clades: (1) *P. pacionii* s. str. and (2) a clade composed of samples found in Iran, as well as one intermediate sequence (GQ228092), which resembles one of these clades in the ITS1 region, and the other in the ITS2 region. This rare case could represent an independent intermediate lineage, a different allele, or a sequencing chimera. Additional data are required to solve this issue and to make the necessary taxonomic updates. Significant intraspecific structures can also be observed in *P. rodensis* and *P. morarae*, and the exact boundaries of both species are similarly uncertain. Even the type, *P. juniperi*, shows a certain variability with two reciprocally monophyletic clades inside it. Additional sequences will show if both are completely isolated (suggesting a very recent speciation event causing “cryptic” species) or else intermediate specimens can be found. If specimens do not group in reciprocally monophyletic clades, specimen AH 39285 could even fall inside a broad concept of *P. juniperi*, as it complies with the characteristic phenotypical features of this species.

High intraspecific genetic variabilities can be found too in the closely related genera *Geopora* s. str. [52,55], *Sepultaria* [48], and *Tricharina* [56], as well as other genera of Pyronemataceae, such as *Genea* [57,58], where some species showed variabilities of 5%, 10%, or even 20% in their ITS rDNA. In some cases, this could be caused by the presence of hidden “cryptic” taxa (as described above), but other species probably have a truly higher-than-average intraspecific variability. This was found to be 2.5% on average in fungal ITS rDNA [59] but between 0 and 1% in most fungal species [60], to the extent that the optimal threshold to separate intra- and interspecific variability is thought to be 99.6% [61]. The higher values found in the present work and those reported above might be due to increased mutation rates, maybe caused by changes in generation times, population size, or even metabolic rates, factors known to modify the appearance of new mutations [62,63].

## Data Availability

The original data presented in the study are openly available in FigShare at dx.doi.org/10.6084/m9.figshare.30998461 (accessed on 8 January 2026).

## Supplementary Materials

The following supporting information can be downloaded at https://www.mdpi.com/article/10.3390/jof12020084/s1: File S1: combined ITS-LSU-RPB2 alignment of *Picoa* and related genera; File S2: ITS rDNA alignment of *Picoa* and *Geopora*; Table S1: Supplementary Table with Genbank accessions of the sequences analyzed.
